# Deletion of the type-1 interferon receptor in APP_SWE_/PS1_ΔE9_ mice preserves cognitive function and alters glial phenotype

**DOI:** 10.1186/s40478-016-0341-4

**Published:** 2016-07-11

**Authors:** Myles R. Minter, Zachery Moore, Moses Zhang, Kate M. Brody, Nigel C. Jones, Sandy R. Shultz, Juliet M. Taylor, Peter J. Crack

**Affiliations:** Department of Pharmacology and Therapeutics, University of Melbourne, 8th floor, Medical building, Grattan St, Parkville, Melbourne, 3010 Victoria Australia; Department of Medicine (The Royal Melbourne Hospital), Melbourne Brain Center, University of Melbourne, Parkville, Melbourne, Victoria Australia

**Keywords:** Alzheimer’s disease, Type-1 interferons, Neuro-inflammation, Cognition, Amyloid-β, Microglial polarization

## Abstract

A neuro-inflammatory response is evident in Alzheimer’s disease (AD), yet the precise mechanisms by which neuro-inflammation influences the progression of Alzheimer’s disease (AD) remain poorly understood. Type-1 interferons (IFNs) are master regulators of innate immunity and have been implicated in multiple CNS disorders, however their role in AD progression has not yet been fully investigated. Hence, we generated APP_SWE_/PS1_ΔE9_ mice lacking the type-1 IFN alpha receptor-1 (IFNAR1, APP_SWE_/PS1_ΔE9_ x IFNAR1^−/−^) aged to 9 months to investigate the role of type-1 IFN signaling in a well-validated model of AD. APP_SWE_/PS1_ΔE9_ x IFNAR1^−/−^ mice displayed a modest reduction in Aβ monomer levels, despite maintenance of plaque deposition. This finding correlated with partial rescue of spatial learning and memory impairments in the Morris water maze in comparison to APP_SWE_/PS1_ΔE9_ mice. Q-PCR identified a reduced type-1 IFN response and modulated pro-inflammatory cytokine secretion in APP_SWE_/PS1_ΔE9_ x IFNAR1^−/−^ mice compared to APP_SWE_/PS1_ΔE9_ mice. Interestingly, immunohistochemistry displayed enhanced astrocyte reactivity but attenuated microgliosis surrounding amyloid plaque deposits in APP_SWE_/PS1_ΔE9_ x IFNAR1^−/−^ mice in comparison to APP_SWE_/PS1_ΔE9_ mice. These APP_SWE_/PS1_ΔE9_ x IFNAR1^−/−^ microglial populations demonstrated an anti-inflammatory phenotype that was confirmed in vitro by soluble Aβ1-42 treatment of IFNAR1^−/−^ primary glial cultures. Our findings suggest that modulating neuro-inflammatory responses by suppressing type-1 IFN signaling may provide therapeutic benefit in AD.

**Electronic supplementary material:**

The online version of this article (doi:10.1186/s40478-016-0341-4) contains supplementary material, which is available to authorized users.

## Introduction

Pathologically, Alzheimer’s disease (AD) is characterized by the extracellular accumulation of Aβ plaques [60] and presence of neurofibrillary tangles containing hyper-phosphorylated tau [22]. Yet targeting these proteinopathies has yet proven clinically efficacious [47]. Neuro-inflammation, involving pro-inflammatory cytokine secretion and reactive gliosis, is evident in AD [34, 37, 38, 48, 54] and epidemiological evidence suggests that this innate immune process is a key contributor to disease pathogenesis [6, 59, 67]. However the exact contribution of cytokines to the exacerbation of neuro-inflammation in AD remains unclear.

Oligomeric and fibrillar Aβ are detected by pattern recognition receptors of the innate immune system and trigger inflammasome activation [57, 58]. Many studies have utilized the APP_SWE_/PS1_ΔE9_ mouse model of AD as a tool to investigate in vivo inflammasome activation in response to Aβ production. These mice displays elevated production of Aβ1-42, leading to progressive plaque deposition and cognitive decline beginning at 6–9 months of age [30, 31]. Removal of the NLRP3 inflammasome, critical for caspase-1-mediated production of IL-1β, in APP_SWE_/PS1_ΔE9_ mice confers reductions in Aβ load, enhances LTP and rescues cognitive impairments [26]. A supportive study addressing the NLRP1 inflammasome also demonstrated that a reduced IL-1β response to amyloid is beneficial in APP_SWE_/PS1_ΔE9_ mice and reduces neuronal pyroptosis [62]. In addition, monoclonal antibodies blocking IL-12/IL-23 signaling attenuate amyloid burden and cognitive defects in APP_SWE_/PS1_ΔE9_ mice [66].

Findings from these studies suggest that microglial phenotype and function play an important role in the exacerbation and progression of AD. Upon stimulus with pro-inflammatory cytokines, microglia can polarize towards a pro-inflammatory phenotype that is deleterious to neurogenesis and synaptic plasticity. Microglial populations can also adopt an alternate anti-inflammatory phenotype that results from exposure to anti-inflammatory cytokines and promotes resolution of inflammation considered to be neuro-protective [45, 55]. These aforementioned studies demonstrate that targeting pro-inflammatory cytokine networks can attenuate neuro-inflammation, promote Aβ clearance and confer cognitive benefit in mouse models of AD by promoting anti-inflammatory activity of microglia. However, Adenoviral delivery of anti-inflammatory cytokines IL-4 or IL-10 results in deleterious effects, impeding Aβ clearance and worsening cognitive decline [10, 11]. In contrast complete removal of IL-10 promotes innate immunity and mitigates AD-like pathology in APP_SWE_/PS1_ΔE9_ mice [23]. Clearly a greater understanding of pro- and anti-inflammatory cytokine signaling is required to explain how modulating innate neuro-inflammation impacts progression of AD.

The pleiotropic type-1 IFNs regulate the aforementioned pro-inflammatory cytokine systems and are master regulators of the innate immune response [19, 33]. By signalling through the type-1 interferon receptor alpha-1 (IFNAR1) and activating the Janus associated kinase/Signal transducer and activator of transcription (JAK/Stat) pathway, type-1 IFNs can induce pro-inflammatory gene transcription generating hallmark cytokines (IL-1β, IL-6 and TNFα) that regulate immune cell recruitment and inflammatory progression. Whilst their contribution to peripheral immunity is well documented, type-1 IFNs are produced and trigger inflammatory cascades in CNS residing neurons and microglia [14, 52]. Elevated type-1 IFN levels have been linked to exacerbation numerous neuro-pathologies including Aicardi-Goutieres syndrome [17, 18] and systemic lupus erythromatosus [8]. It is now considered that a balance of interferon signalling is required for healthy brain physiology and dysregulation of this cytokine system can result in brain ‘interferonopathies’ [20]. Significantly, an exacerbated type-1 IFN response contributes to many deleterious effects associated with the aging process [3]. In addition, a type-1 IFN signature in both human AD patients and APP_SWE_/PS1_ΔE9_ mice is evident and removal IFNAR1 confers protection against soluble Aβ1-42-induced toxicity in primary cultured neurons [64].

We hypothesize that removal of type-1 IFN signaling attenuates neuro-inflammation and delays phenotypic progression in APP_SWE_/PS1_ΔE9_ mice. To test this, we generated APP_SWE_/PS1_ΔE9_ x IFNAR1^−/−^ mice and used primary mixed astrocyte and microglial cultures and primary neuronal cultures to investigate the role of type-1 IFN signaling in AD. We find that APP_SWE_/PS1_ΔE9_ x IFNAR1^−/−^ mice display modest reductions in monomeric Aβ load, without altering plaque deposition, and improved spatial cognitive performance. These mice exhibit a predominantly anti-inflammatory glial phenotype when compared to APP_SWE_/PS1_ΔE9_ mice alone. We confirm this anti-inflammatory glial phenotype in vitro in response to Aβ1-42 and demonstrate this polarization state protects primary neuronal cultures. Importantly, this study demonstrates that removal of type-1 IFN signaling modulates neuro-inflammation and retards phenotypic progression in the APP_SWE_/PS1_ΔE9_ mouse model of AD.

## Materials and Methods

### Animals

APP_SWE_/PS1_ΔE9_ transgenic mice [30] on a C57BL/6 background were sourced from JAX. (B6.Cg-Tg(APPswe,PSEN1dE9)85Dbo/Mmjax, JAX ID: 005864, https://www.jax.org/strain/005864). IFNAR1^−/−^ mice on a C57Bl/6 background were initially generated by [28]. APP_SWE_/PS1_ΔE9_ transgenic mice lacking IFNAR1 were generated by interbreeding. APP_SWE_/PS1_ΔE9_ and IFNAR1^−/−^ mice to produce F1 progeny. APP_SWE_/PS1_ΔE9 x_ IFNAR1^+/−^ mice from F1 progeny were then interbred to yield APP_SWE_/PS1_ΔE9_ x IFNAR1^−/−^ mice (F2 progeny). In all animal experiments, mice were used at 9 months of age with aged-matched littermate non-transgenic wildtype and IFNAR1^−/−^ control mice. All mice were determined as specific pathogen-free, housed in sterile micro-isolator cages and fed ad-libitum on standard chow with open access to water. All animal procedures were performed in accordance with the University of Melbourne animal care committee’s regulations.

### Animal genotyping

All animals used in this study were analysed for correct genotypes before use. This genotyping was either performed manually (Additional file 1: Figure S1), as described below, or in partnership with Transnetyx™ (Cordova, TN, USA).

Tails from mice were obtained pre-weaning and genomic DNA was extracted. Tissue was digested using Proteinase K (9.3 mg/ml, New England Biolabs) in Tris buffer (containing 1 % w/v SDS). Upon removal of protein with Potassium acetate (1.5 M) and precipitation using propan-2-ol, the extracted DNA was washed in 70 % Ethanol and reconstituted in Tris-EDTA (TE) buffer. PCR was then conducted using the GoTaq® DNA polymerase system (M3005, Promega) under the following conditions:

**Table:** 

Step #	Temperature (°C)	Time (seconds)	Comments
1	94	180	
2	94	30	X35 repeats
3	54	60	
4	72	60	
5	72	120	
6	4	∞	Holding temperature

PCR products were then loaded into a 2 % w/v agarose Et-Br-labelled (40 μg/ml) gel and electrophoresis was performed to separate bands. A pre-stained BenchTop 100 bp DNA ladder (G8291, Promega) was used to determine sample band sizes upon gel imaging using the ChemiDoc™ MP image system (Biorad). Specific oligonucleotide primers used for amplification of APP_SWE_, PS1_ΔE9_, IFNAR1 and internal control DNA sequences are detailed below:

**Table:** 

Primer name	Direction (5’ → 3’)	Sequence (5’ → 3’)	Reaction concentration
oIMR3610 (APP)	Forward	AGG ACT GAC CAC TCG ACC AG	1 μM
oIMR3611 (APP)	Reverse	CGG GGG TCT AGT TCT GCA T	1 μM
oIMR1644 (PS1)	Forward	AAT AGA GAA CGG CAG GAG CA	1.33 μM
oIMR1645 (PS1)	Reverse	GCC ATG AGG GCA CTA ATC AT	1.33 μM
oIMR7338 (Control)	Forward	CTA GGC CAC AGA ATT GAA AGA TCT	0.5 μM
oIMR7339 (Control)	Reverse	GTA GGT GGA AAT TCT AGC ATC ATC C	0.5 μM
mIFNAR1E4F (IFNAR1)	Forward	CTC CTC CCG GAC AAG ACG GG	1 μM
mIFNAR1E5R (IFNAR1)	Reverse	TGG TGC TTA TAC ACT GCA CAG TGC T	1 μM
NeoF (neomycin)	Forward	GAG GCA GCG CGG CTA TCG TG	0.5 μM

### In vivo study structure

The number of mice used in this study were as follows: Wildtype: 15; IFNAR1^−/−^: 18; APP_SWE_/PS1_ΔE9_; APP_SWE_/PS1_ΔE9_ x IFNAR1^−/−^: 9. Both male and female mice were used in the study in 50:50 proportion. No significant sex difference was detected in behavior and/or biochemical readouts and thus sexes were pooled. All mice were subjected to Morris water testing then 9 mice from each genotype were randomly selected for biochemical processing. Half hemispheres were taken for immunohistochemistry, whilst the other half was snap frozen and ground on liquid nitrogen in order to isolate both protein and RNA from the same mice.

### Mixed cortical and hippocampal glial isolation

Mixed cortical and hippocampal neurons were isolated from embryonic P0-1 embryos as previously described [35]. Briefly, cortices were isolated and meninges surgically removed. The cleaned cortical tissue was then digested in hanks buffered sulphate solution (HBSS, 14025–092, Gibco) using trypsin/DNAse (1 mg/ml, T9201, D5025, Sigma) until a single cell suspension was achieved. Cells were then plated at a density of 1 brain/10 ml in culture medium (DMEM, 31985–062, Gibco, 20 % FBS, 0.5 % penicillin-streptomycin) in T75cm^2^ flasks. Media was then replaced every two days until the glial cultures formed a comprehensive monolayer. Cells were seeded at 5 x 10^5^ cells/ml for experimental use between 14 and 28 days in vitro.

### Mixed cortical and hippocampal neuron isolation

Mixed cortical and hippocampal neurons were isolated as described previously [63]. Briefly, cortices were isolated from embryonic day 14–16 pups and meninges were removed. Cleaned cortices were then digested in Krebs solution containing trypsin (250 μg/ml) and DNAse (33 μg/ml) and mechanically agitated to ensure a single cell suspension. Cells were then plated into pre-coated Poly-L-lysine (0.5 mg/ml, P6282, sigma) plastic ware at a density of 1 x 10^6^ cells/ml in neurobasal media (17504–044, Gibco) containing B-27 growth factor supplement (17504–044, Gibco), L-glutamine (500 μM, G7513, Sigma) and 2 % FBS. The following day, FBS was removed from the media and cultures were supplemented with fresh culture media every two days until experimental use. Neuronal purity was assessed at >90 % using NeuN (neuronal nuclei) immunohistochemistry (data not shown) and all cultures were used day 9–10 in vitro.

### Cardiac perfusion of mice and isolation of brain tissue

Mice were deeply anaesthetized using intra-peritoneal injection of combinatorial ketamine (90 mg/kg) and xylazine (4.5 mg/kg, K113, Sigma). Mice were cardiac perfused with ice-cold heparinized PBS (1U/ml, H3393, Sigma). Brains were then excised and separated for use in immunohistochemistry or for RNA/protein biochemical analysis. Isolation of the cortex for RNA/protein biochemistry was performed using a modified dissection technique [24]. Hemispheres were placed on an ice cold glass dissection plate and orientated in a sagittal plane. The cerebellum was removed, and the striatum, thalamus, midbrain and brain stem remnants were identified. These structures were then removed using sterilized blunt spatulas, exposing the hippocampal complex and interior wall of the cortex. The hippocampus was then peeled away from the cortex, and cortical tissue was snap frozen in liquid nitrogen and stored at −80 °C until required.

### Immunohistochemistry

For immunohistochemical analysis, hemispheres were post-fixed in 4 % w/v paraformaldehyde in PBS for 72 h (4 °C) before being transferred into 30 % w/v sucrose in PBS for 48 h (4 °C) and embedded in Optimal Cutting Temperature (OCT, 4583, Sakura) medium for subsequent cryosectioning. Sagittal sections (30 μm) were then cut throughout the hippocampal region using a cryostat (Reichert-Jung) and mounted onto electrostatic Menzel-Gläser Superfrost® plus glass microscopy slides (J1800AMNZ, Thermo-Scientific). Tissue was then permeabilized in PBS-T (0.05 % v/v Tween-20, 5 min, room temperature) and blocked in CAS-Block™ (1 h, room temperature, 008120, Invitrogen). After rinsing with PBS, tissue was then incubated overnight with primary antibody diluted in 10 % v/v CAS-Block™ in PBS (4 °C, humidified chamber). After washing in PBS, slides were then incubated with fluorescent secondary antibodies diluted in 10 % v/v CAS-Block™ in PBS (2 h, room temperature). Post-PBS rinse coverslips were mounted in Vectashield® DAPI-containing mounting media (H-1200, Vector laboratories). Images were then obtained using a Zeiss Axio Observer.Z1 (Carl Zeiss imaging) inverted fluorescence microscope. Details of antibodies used for immunohistochemistry are provided below:

**Table:** 

Antibody	Supplier	Source	Dilution
GFAP	Dako (Z0334)	Rabbit pAb	1:1000
IBA-1	Wako (019–19741)	Rabbit pAb	1:300
WO-2	(Wun et al., 2008 [68])	Mouse mAb IgG_1_	1:500
Alexa Fluor® 488 Goat anti-rabbit	Molecular probes (A-11008)	Goat pAb	1:1000
Alexa Fluor® 594 Goat anti-mouse	Molecular probes (A-11005)	Goat pAb	1:1000

To quantify plaque number and burden, WO-2 immunofluorescent labelled sections were converted to 8-Bit images and an image fluorescence threshold was set using Image J quantification software (NIH). Plaque staining was then analysed by particle quantification giving plaque number. The WO-2 positive pixel coverage value was then expressed relative to total cortical area to calculate cortical plaque burden. For IBA-1 and GFAP immunofluorescence quantification, integrated densities were calculated from entire cortical regions using Image J quantification software (National Institutes of Health, NIH). These values were then normalized to staining background and expressed as relative fluorescence intensity as described previously [32]. All quantified data is from average values generated from ≥3 sections/mouse (each 270 μm apart or every 6th section).

### Amyloid beta preparation and cell culture treatment

Amyloid peptide stocks were prepared according to methods described previously [2]. Aβ1-42 stocks (A-42-T-1, GenecBio) were initially monomerized in 1, 1, 1, 3, 3, 3-Hexafluoro-2-propanol (0.5 mg/ml, HFIP, 52512, Fluka), lyophilized and stored at −80 °C until required. The peptide was then dissolved in ice cold 5 mM NaOH in Dulbecco’s PBS by rigorous vortexing and protein concentration was determined by absorbance spectrophotometry at 214 nm. Peptide concentrations were calculated using Eq. 1.

1 $$ \left[A\beta 42\right] = Ab{s}_{214}x\ \left(DF/\varepsilon \right) $$

Where Abs_214_ = Absorbance value of sample at 214 nm, DF = Sample dilution factor, ε = molar extinction coefficient of Aβ42 (75,887 L/mol/cm).

Primary cultured glial cells were treated with 10 μM Aβ1-42 or NaOH vehicle for up to 96 h in serum-reduced glial treatment medium (DMEM with 2 % FBS and 0.5 % penicillin-streptomycin). The final concentration of NaOH across all treatment groups was <5nM and remained non-toxic.

### Protein extraction

Following treatment, primary glial cell cultures were washed in ice cold PBS and collected via cell scraping. Cell pellets were briefly sonicated in Tris lysis buffer (50 mM Tris, 150 mM NaCl, 1 % v/v Triton x-100 (T8787, Sigma), 1 % w/v SDS, PhosphoSTOP® phosphatase and cOmplete® protease inhibitors (04906837001, 11697498001, Roche), pH 7.4). Brain tissue was homogenized in Tris lysis buffer (≤100 mg/ml concentration, without 1%w/v SDS). Upon rotation for 90 min at 4 °C, homogenates were centrifuged (12,000xg, 4 °C, 5 min) before supernatants were removed and stored at −80 °C until required. Protein concentrations were determined as per the method of Bradford ([5], 500–0006, Bio-Rad).

### SDS-PAGE gel electrophoresis and Western blotting

Fifty micrograms of protein was denatured in reducing buffer (20 mM Tris, 20%v/v glycerol, 4%w/v SDS, 10 % β-mecaptoethanol (M6250, Sigma), and bromophenol blue). Samples were loaded onto 10 % SDS-PAGE gels (60 mM Tris, 0.1 % w/v SDS, 0.1 % w/v APS, 0.01 % v/v TEMED, 10 % Acrylamide/Bis (161–0156, Bio-Rad)) or 4-20 % Mini-PROTEAN® TGX Stain-Free™ gels (456–8093, Bio-Rad) and electrophoresis was performed at 120 V in Novex® Tris-Glycine SDS running buffer (LC2675-5, Invitrogen). Proteins were then transferred to polyvinylidene fluoride (PVDF) membranes by semi-dry transfer (60 mA/gel, 1.25 h) or using the TransBlot® Turbo™ transfer system (2.5MA, 7 min, 170–4155, Bio-Rad) as per manufacturer’s instructions. Membranes were blocked in 5%w/v skim milk powder in Tris buffered saline-Tween 20 (0.05 % v/v Tween-20, TBS-T) for 1 h before overnight incubation with primary antibodies at 4 °C (dilutions in 2 % w/v skim milk powder in TBS-T). Membranes were then washed in TBS-T before being incubated with horseradish peroxidase (HRP)-conjugated secondary antibodies (dilutions in 2 % w/v skim milk powder in TBS-T) for 90 min at room temperature. HRP signals were detected using an enhanced chemiluminescence (ECL™) prime Western blotting detection kit (RPN2232, Amersham) and visualized using the ChemiDoc™ MP system (Bio-Rad). For densitometry analysis, all raw pixel intensities of HRP signals from Western blots were calculated using Image J quantification software (NIH). Details of antibodies used for Western blotting are provided below:

**Table:** 

Antibody	Supplier	Source	WB dilution
p-Stat-3 (Y705)	Cell Signaling (9145)	Rabbit mAb IgG_1_	1:1000
Stat-3	Cell Signaling (9132)	Rabbit pAb	1:1000
WO-2	[68]	Mouse mAb IgG_1_	1:2000
p-NFkB (p65, S536)	Cell Signaling (3033)	Rabbit mAb IgG_1_	1:1000
SOCS3	Abcam (ab723)	Goat pAb	1:200
β-actin	Sigma-Aldrich (A5441)	Mouse mAb IgG_1_	1:4000
G α-rabbit/HRP	Dako (P0448)	Goat pAb-HRP	1:1000
G α-mouse/HRP	Dako (P0447)	Goat pAb-HRP	1:1000

### Aβ1:40 sandwich Enzyme-linked immunosorbent assay (ELISA)

For quantification of soluble and insoluble Aβ levels, tissues were homogenized in PBS (containing Triton x-100, 1 % v/v, PBS-T) containing PhosphoSTOP® phosphatase and cOmplete® protease inhibitors (04906837001, 11697498001, Roche), pH 7.4) via sonication. After centrifugation (100,000xg, 60 min, 4 °C) the supernatant was collected to detect PBS-T-soluble Aβ1:40 levels. The remaining tissue pellets were further homogenized in 70 % v/v formic acid in PBS, centrifuged (100,000xg, 60 min, 4 °C) and the resulting supernatant was neutralized with 1 M Tris base (20x volume) and collected to detect PBS-T-insoluble Aβ1:40 levels by sandwich ELISA.

Assays were run in 96-well plate format with all standards and samples run in duplicate reactions. ELISA plates were coated in WO-2 capture antibody (diluted in 0.05 M carbonate-bicarbonate, pH 9.6) overnight and blocked in 1 % BSA (diluted in TBS-T) prior to sample incubation (100 μg protein/well, 4 °C, overnight incubation with shaking). Plates were then washed in TBS-T and incubated with an anti-Aβ1:40 casein-1E8 biotinylated monoclonal sandwich detection antibody. After washing in TBS-T plates were incubated with high sensitivity Streptavidin-HRP and signals were developed using TMB substrate and detection at 450 nm. Sample absorbance were normalized to the Aβ1:40 standard curve and concentrations are expressed relative to sample total protein concentrations as determined by Bradford assay.

### RNA isolation and cDNA synthesis

RNA was extracted from cell pellets or brain tissue by methods previously described [12] using TRIzol® reagent (15596018, Life-Technologies). Contaminating genomic DNA was then removed prior to reverse transcription using the TURBO DNA-*free*™ kit (AM1907, Ambion) according to manufacturer’s guidelines. Yield quantities and purity of the RNA product was then assessed using the nanodrop 1000 spectrophotometer (Thermo Scientific). One microgram of RNA was then reverse transcribed to produce cDNA using a high capacity cDNA reverse transcription kit (4368814, Applied Biosciences) as per manufacturer’s instructions and cDNA was then diluted 1:3 in diethylpyrocarbonate (DEPC)-treated dH_2_O for use in QPCR.

### Quantitative PCR

All QPCR was performed in standard 384-well plates (4309849, Applied Biosystems) using the 7900ht fast real-time PCR system (Applied Biosystems) and reactions for a given sample were performed in triplicate. All Taqman gene expression assays were purchased commercially (431182, Applied Biosystems) and reactions were performed under the following thermal conditions:

**Table:** 

Step #	Temperature (°C)	Time (minutes)	Comments
1	50	2	-
2	94.5	10	-
3	97	0.5	X40 repeats
4	59.7	1	

For SYBR® green-based detection, gene-specific primers were synthesized commercially (Geneworks) and reactions were performed under the following thermal conditions:

**Table:** 

Step #	Temperature (°C)	Time (minutes)	Comments
1	95	20	-
2	95	0.5	X40 repeats
3	60	1.5	
4	95	15	-
5	60	15	-
6	95	15	-

Fold change readouts presented throughout the study were calculated using the ΔΔct calculation method [36]. For each experiment a fluorescence detection threshold was automatically set at 1.0 RFU and the cycle number at which each reaction reached this threshold was calculated (cycle threshold (Ct)). Triplicate Ct value for genes of interest were then normalized back to the Ct values of the GAPDH housekeeping gene to account for differences in original cDNA concentration between samples (ΔCt). The calculated ΔCt of treatment or genotype groups were then normalized back to the ΔCt of appropriate genotype-specific control samples. In addition, ΔΔCt values were converted to fold change data using Eq. 2.

2 $$ Fold\  change = {2}^{\left(-\varDelta \varDelta ct\right)} $$

Primers used for QPCR analysis are listed below:

**Table:** 

Gene	Species	Inventory number
GAPDH	Mouse	Mm99999915_m1
IFNβ	Mouse	Mm00439552_s1
IRF7	Mouse	Mm00516788_m1
IRF3	Mouse	Mm00516779_m1
IRF8	Mouse	Mm00492567_m1
IL-1β	Mouse	Mm01336189_m1
IL-6	Mouse	Mm00446190_m1
TNFα	Mouse	Mm00443258_m1
CD33	Mouse	Mm00491152_m1
TREM2	Mouse	Mm04209422_m1

**Table:** 

Gene	Direction (5’ → 3’)	Sequence (5’ → 3’)
GAPDH	Forward	ATCTTCTTGTGCAGTGCCAGC
	Reverse	ACTCCACGACATACTCAGCACC
IFNα	Forward	GCAATCCTCCTAGACTCACTTCTGCA
	Reverse	TATAGTTCCTCACAGCCAGCAG
IFNαE4	Reverse	TATTTCTTCATAGCCAGCTG
iNOS	Forward	CAAGCACCTTGGAAGAGGAG
	Reverse	AAGGCCAAACACAGCATACC
CD32	Forward	AATCCTGCCGTTCCTACTGATC
	Reverse	GTGTCACCGTGTCTTCCTTGAG
CD11b	Forward	CCAAGACGATCTCAGCATCA
	Reverse	TTCTGGCTTGCTGAATCCTT
CD206	Forward	CAAGGAAGGTTGGCATTTGT
	Reverse	CCTTTCAGTCCTTTGCAAGC
ARG1	Forward	TCACCTGAGCTTTGATGTCG
	Reverse	CTGAAAGGAGCCCTGTCTTG
CCL22	Forward	CTGATGCAGGTCCCTATGGT
	Reverse	GCAGGATTTTGAGGTCCAGA
TGFβ	Forward	TGCGCTTGCAGAGATTAAAA
	Reverse	CGTCAAAAGACAGCCACTCA
YM1	Forward	CAGGGTAATGAGTGGGTTGG
	Reverse	CACGGCACCTCCTAAATTGT

### MTS cell viability assay

Cell viability was measured by the ability to metabolize 3-(4,5-dimethylthiazol-2-yl)-5-(3-carboxymethoxyphenyl)-2-(4-sulfophenyl)-2H-tetrazolium (MTS, CellTiter 96® AQ_ueous_ non-radioactive cell proliferation assay, G5421, Promega) in the presence of the electron coupler phenazine methosulfate (PMS) to a media soluble formazan product, as described previously [9]. Combined MTS (400 μg/ml) and PMS (44 μg/ml) solution was incubated on primary cultured neurons for 4 h (37 °C). Culture medium absorbance at 492 nm was then determined using a Multiskan Ascent spectrophotometer (Thermo Scientific). All sample absorbance readings were then blank normalized using a negative control reaction containing only culture medium and the MTS/PMS reagent. Absorbance readings of all treatment groups were then normalized back to genotype-specific vehicle controls and expressed as a percentage of vehicle control cell viability. Experiments were performed in technical triplicate and staurosporine (1 μM, S6942, Sigma) was used to induce cellular apoptosis in primary neuronal cultures for positive control means.

### Morris water maze

Morris water maze (MWM) testing was conducted in a black circular pool 1.6 m in diameter and 0.8 m deep. A white Perspex 10 cm^2^ circular platform was then secured to the enclosure 25 cm from the pool wall. The pool was then filled with water at 21–23 °C until the white platform was submerged 1 cm below the surface. Non-toxic, water soluble white ceiling paint (Taubmans) was then used to opacify the water. The room was brightly illuminated using wall-mounted halogen lamps. Several distinct and distal extra-maze cues were placed around the pool as points of reference. These cues remained in place throughout the duration of testing and the platform was only removed to conduct the probe trial.

Mice were subjected to a 4 trial/day protocol for 7 days with 60 s maximum trial duration. Mice were removed from their test cage and placed into the water maze, at a randomized cardinal point, facing towards the pool wall. The 60 s trial commenced after the mouse had entered the MWM for 2 s. If the test mouse found, mounted and stayed on the hidden platform for 2 s the trial was deemed complete and latency to reach the platform was calculated. The mouse remained on the hidden platform for 20 s before being removed from the water maze and placed back in their testing cage. If the mouse failed to find or remain on the platform for at least two seconds before the 60 s time allowance, the researcher entered the testing area and guided the mouse to the hidden platform. Once an individual mouse completed the trial and was towel dried, the next mouse within the testing group was immediately placed into the MWM for testing. Each mouse underwent 4 trials per day, according to the aforementioned trial parameters and had an inter-trial resting time of 10 min. All MWM testing was recorded to DVD and automatically tracked using Ethovision® XT (Noldus). For all individual trials, latency to platform, success rate, path length and swim speed was calculated. A 60 s value for latency was awarded for all trials where the test mouse failed to find the platform within the allocated time.

To assess spatial reference memory, a probe trial was conducted on day 7 of MWM acquisition. Mice were placed into the maze at the north cardinal point and allowed to explore for the standard 60 s trial length with the escape platform removed. The maze was virtually divided up into quadrants and time spent in the quadrant which previously held the platform was calculated. All primary readouts reported from MWM testing conducted in this study are well-established in the AD field [7].

### Statistical analysis

GraphPad Prism software (version 6.0, http://www.graphpad.com/scientific-software/prism/) was used for all *t*-tests, ANOVAs and post-hoc statistical evaluation. Where comparisons of multiple groups was required a one or two-way analysis of variance (ANOVA) was performed, with mouse genotype as the fixed variable. A Bonferroni post-hoc or Tukey’s HSD multiple comparisons test was then performed. Otherwise an unpaired two-tailed Student’s *t*-test was used. For all statistical tests a two-tailed α value of 0.05 was utilized. Box plots were used to display data in which the midline represents the median value and the upper and lower margins equate to the 25 % and 75 % quartiles. The whiskers display data within the 1.5xinterquartile range and values beyond this were determined as outliers (represented as circles). All other numerical data is presented as mean ± SEM. Power values for each test where calculated post-hoc using G*Power (version 3.1, http://gpower.hhu.de/), based upon the effect size, group number and sample size. Exact p-values were calculated for all Student’s *t*-tests and multiplicity adjusted p-values were determined for all Bonferroni’s and Tukey’s post-hoc tests. A p-value <0.05 was considered statistically significant. All use of statistics is detailed in Additional file 2: Table S1.

## Results

### Removal of IFNAR1 in APP_SWE_/PS1_ΔE9_ mice confers modest reductions in cortical Aβ monomer load but plaque burden remains unaltered

**Fig. 1:**
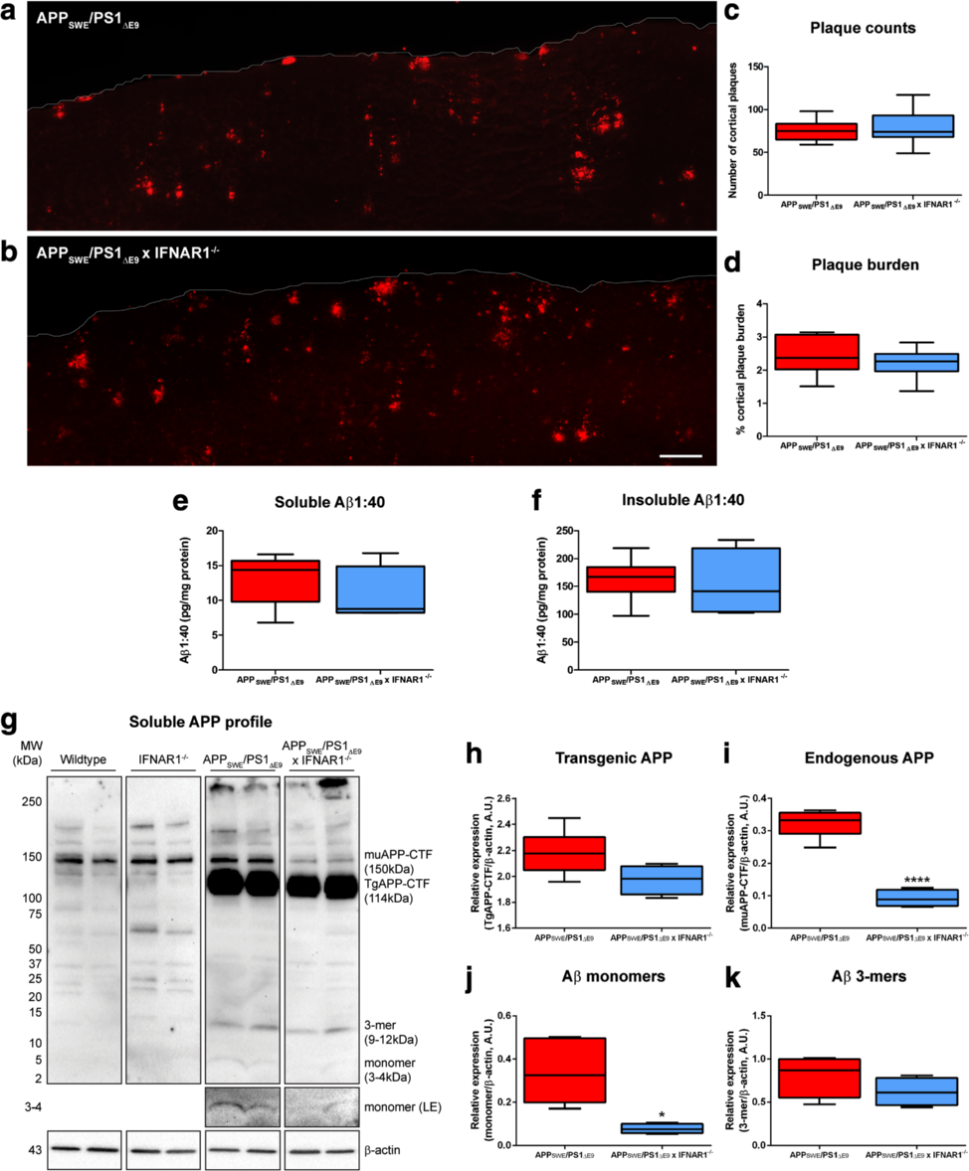
Removal of IFNAR1 in APP_SWE_/PS1_ΔE9_ mice confers modest reductions in Aβ monomer levels but not plaque burden. Representative cortical sections from 9 month old **a** APP_SWE_/PS1_ΔE9_ and **b** APP_SWE_/PS1_ΔE9_ x IFNAR1^−/−^ mice stained with anti-Aβ mAb WO-2 using fluorescence immunohistochemistry (scale bar = 200 μm). **c** Aβ plaques were counted from entire cortical regions of APP_SWE_/PS1_ΔE9_ and APP_SWE_/PS1_ΔE9_ x IFNAR1^−/−^ mice (3 sections per mouse, •represents outlier value). **d** Cortical plaque burden was calculated by quantifying Aβ plaque immunofluorescence relative to total cortical area from these same cortical slices of APP_SWE_/PS1_ΔE9_ and APP_SWE_/PS1_ΔE9_ x IFNAR1^−/−^ mice. **e** PBS-T-soluble and **f** PBS-T-insoluble Aβ1:40 levels in APP_SWE_/PS1_ΔE9_ and APP_SWE_/PS1_ΔE9_ x IFNAR1^−/−^ mouse cortical lysates were quantified by ELISA. **g** Representative immunoblot of Tris–HCl soluble cortical protein lysates isolated from 9 month old wildtype, IFNAR1^−/−^, APP_SWE_/PS1_ΔE9_ and APP_SWE_/PS1_ΔE9_ x IFNAR1^−/−^ mice using the anti-Aβ mAb WO-2. Multiple amyloid species can be detected including endogenous APP-CTF (muAPP-CTF), transgenic APP-CTF (TgAPP-CTF), Aβ trimers (3-mer) and Aβ monomers. A long exposure (LE) was used to enhance detection of Aβ monomer levels. Densitometry of **h** Transgenic APP-CTF, **i** endogenous murine APP-CTF, **j** Aβ monomer and **k** 3-mer levels in APP_SWE_/PS1_ΔE9_ and APP_SWE_/PS1_ΔE9_ x IFNAR1^−/−^ mice is shown. All densitometry is expressed as a ratio of Aβ monomer:β-actin or Aβ trimer:β-actin raw pixel intensities. Immuno-detection of β-actin was used to ascertain loading quantities. Data is presented as box plots described in the statistical analysis section in Materials and Methods (immunohistochemistry: *n* = 9 per genotype; ELISA and Western blotting: *n* = 6 (APP_SWE_/PS1_ΔE9_), *n* = 4 (APP_SWE_/PS1_ΔE9_ x IFNAR1^−/−^); *p < 0.05, ****p < 0.0001). See Additional file 2: Table S1 for further analysis

To investigate the effect of removing type-1 IFN signaling in AD we generated APP_SWE_/PS1_ΔE9_ x IFNAR1^−/−^. APP_SWE_/PS1_ΔE9_ mice aged 9 months display an enhanced type-1 IFN and pro-inflammatory cytokine response [64]. Hence, we focused on characterizing phenotypic alterations in APP_SWE_/PS1_ΔE9_ x IFNAR1^−/−^ mice at this age. With variable hippocampal Aβ plaque deposition at this age in both APP_SWE_/PS1_ΔE9_ and APP_SWE_/PS1_ΔE9_ x IFNAR1^−/−^ mice (data not shown), the current study focused on cortical regions only, not hippocampus. To assess potential alterations in Aβ plaque burden, immunohistochemistry was performed on APP_SWE_/PS1_ΔE9_ and APP_SWE_/PS1_ΔE9_ x IFNAR1^−/−^ mouse brain sagittal sections, stained with anti-Aβ mAb WO-2 (*n* = 9 per genotype, Fig. 1a, b). Both APP_SWE_/PS1_ΔE9_ and APP_SWE_/PS1_ΔE9_ x IFNAR1^−/−^ mice display extensive plaque deposition within cortical regions but no difference was detected between genotypes when Aβ plaques were counted (*n* = 9 per genotype, Fig. 1c) or when cortical plaque burden percentage was quantified (*n* = 9 per genotype, Fig. 1d). To validate these immunohistochemical findings we prepared PBS-T-soluble and PBS-T-insoluble fractions from cortical tissue to quantify Aβ levels by ELISA. We did not observe any differences in PBS-T-soluble or PBS-T-insoluble Aβ1:40 levels measured from cortical tissue lysates of APP_SWE_/PS1_ΔE9_ and APP_SWE_/PS1_ΔE9_ x IFNAR1^−/−^ mice (*n* = 4–6 per genotype, Fig. 1e, d). These findings suggest that removal of type-1 IFN signaling in APP_SWE_/PS1_ΔE9_ mice does not influence amyloid plaque deposition at 9 months of age.

Whilst amyloid plaque levels remained unchanged in the APP_SWE_/PS1_ΔE9_ IFNAR1^−/−^ mouse, the oligomerization state of soluble Aβ species may be altered. This can influence peptide toxicity and potentially impact cognitive phenotypes [1, 15, 44]. To investigate the oligomerization state of various Aβ species, we analyzed Tris–HCl soluble cortical fractionations from wildtype, IFNAR1^−/−^, APP_SWE_/PS1_ΔE9_ and APP_SWE_/PS1_ΔE9_ x IFNAR1^−/−^ mice by western blotting, probed with anti-Aβ mAb WO-2 (*n* = 4–6 per genotype, Fig. 1g). Analysis of Aβ oligomers in wildtype and IFNAR1^−/−^ mice displayed constitutive Aβ production but not overexpression that is characteristic of the APP_SWE_/PS1_ΔE9_ transgene. Densitometry identified a trend, albeit not statistically significant, to decreased transgenic human APP-CTF expression (*n* = 4–6 per genotype, *p =* 0.0618, Fig. 1h) and significant reductions in endogenous murine APP-CTF levels (*n* = 4–6 per genotype, *p <* 0.0001, Fig. 1i) in APP_SWE_/PS1_ΔE9_ x IFNAR1^−/−^ mice compared to APP_SWE_/PS1_ΔE9_ mice. Densitometry confirmed a significant 4.3 ± 0.2-fold decrease of cortical Aβ monomer levels in APP_SWE_/PS1_ΔE9_ x IFNAR1^−/−^ mice compared to APP_SWE_/PS1_ΔE9_ mice (*n* = 4–6 per genotype, *p =* 0.0122, Fig. 1j). Although not statistically significant, Aβ trimer (3-mer) levels also trended to a decrease in APP_SWE_/PS1_ΔE9_ x IFNAR1^−/−^ mice compared to APP_SWE_/PS1_ΔE9_ mice (*n* = 4–6 per genotype, *p =* 0.0569, Fig. 1k). Collectively, this data highlights that removal of IFNAR1 in APP_SWE_/PS1_ΔE9_ mice does not influence Aβ plaque deposition, but may influence oligomerization through modest, but significant, reductions in Aβ monomer levels.

### Spatial learning and memory defects in APP_SWE_/PS1_ΔE9_ mice are improved upon removal of IFNAR1

**Fig. 2:**
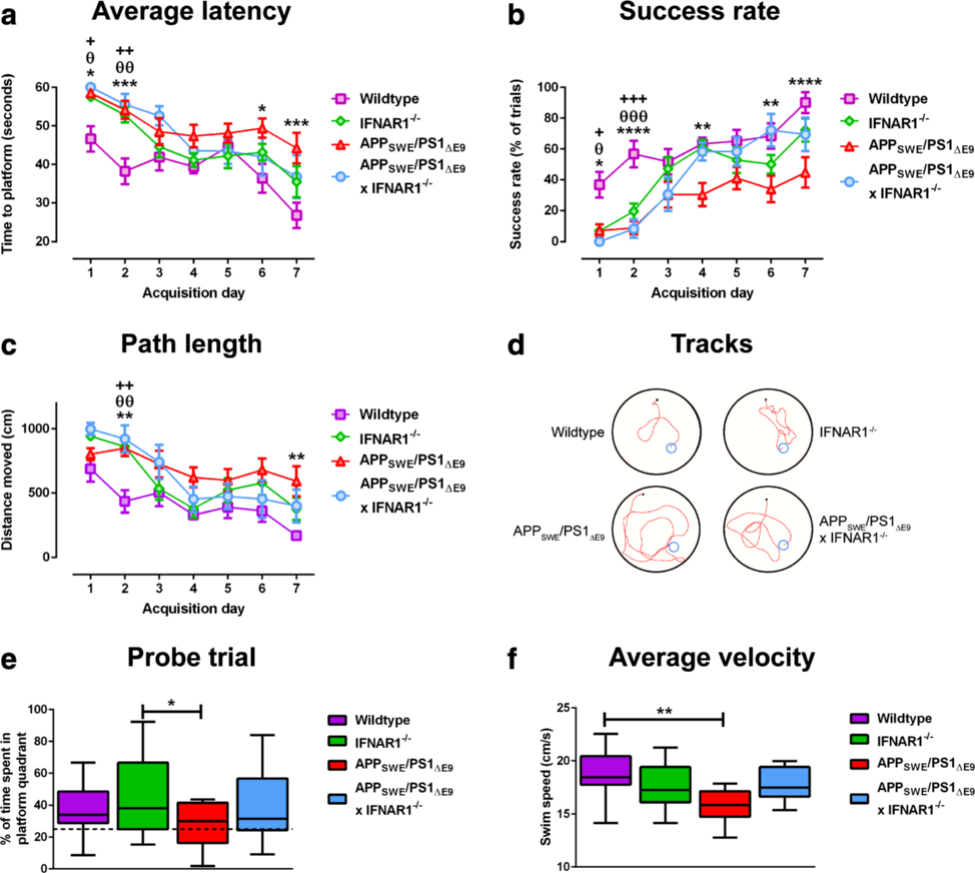
APP_SWE_/PS1_ΔE9_ x IFNAR1^−/−^ mice are spared from cognitive impairment. Wildtype, IFNAR1^−/−^, APP_SWE_/PS1_ΔE9_ and APP_SWE_/PS1_ΔE9_ x IFNAR1^−/−^ littermate controls 9 months of age were subjected to Morris water maze testing to assess spatial learning and memory. All mice were tested using a 7-day hidden platform acquisition (4trials/day) with probe trial protocol as described in Materials and Methods. Primary water maze readouts of **a** average trial latency, **b** trial success rate and **c** trial path length (*p < 0.05, **p < 0.01, ***p < 0.001, ****p < 0.0001, WT vs. APP_SWE_/PS1_ΔE9_; θ*p <* 0.05, θθ*p <* 0.01, θθθ*p <* 0.001, θθθθ*p <* 0.0001, WT vs. APP_SWE_/PS1_ΔE9_ x IFNAR1^−/−^; +*p <* 0.05, ++*p <* 0.01, +++*p <* 0.001 WT vs. IFNAR1^−/−^). **d** Representative automated tracks from day 7 testing is shown for all genotypes. **e** After day 7 acquisition the escape platform is removed from the maze and mice are introduced into the maze for a final trial. Quantification of the time spent in the platform containing quadrant for all genotypes is shown. The dashed line (y = 25 %) represents the percentage of time spent in the escape quadrant that would be solely due to random chance as opposed to preference (*p <* 0.05). **f** Calculation of average swim velocity across all 7 days of testing is shown for all genotypes (**p < 0.01). Data is displayed as mean ± SEM or box plots described in the statistical analysis section in Materials and Methods (*n* = 14 (APP_SWE_/PS1_ΔE9_), *n* = 9 (APP_SWE_/PS1_ΔE9_ x IFNAR1^−/−^), *n* = 18 (IFNAR1^−/−^), *n* = 15 (wildtype)). See Additional file 2: Table S1 for further analysis

To assess if removal of type-1 IFN signaling can alleviate the cognitive deficits observed in APP_SWE_/PS1_ΔE9_ we analyzed spatial learning and memory performance of wildtype, IFNAR1^−/−^, APP_SWE_/PS1_ΔE9_ and APP_SWE_/PS1_ΔE9_ x IFNAR1^−/−^ mice using the Morris water maze. Compared to wildtype, APP_SWE_/PS1_ΔE9_ mice required more time to find the escape platform, whilst APP_SWE_/PS1_ΔE9_ x IFNAR1^−/−^ mice were initially impaired but recovered to wildtype levels as pheno-copied by IFNAR1^−/−^ mice (*n* = 9–18 per genotype, 0.05 < *p <* 0.001, Fig. 2a). Compared to wildtype, an initial decline in trial success rate was seen for all genotypes but this was only maintained by the APP_SWE_/PS1_ΔE9_ mice over the course of acquisition (*n* = 9–18 per genotype, 0.05 < *p <* 0.0001, Fig. 2b). Compared to wildtype, all genotypes initially selected longer escape paths but only APP_SWE_/PS1_ΔE9_ mice maintained this abnormality throughout testing (*n* = 9–18 per genotype, *p <* 0.01, Fig. 2c). Representative tracks (Day 7 acquisition) of APP_SWE_/PS1_ΔE9_ mice display a lack of cue-directed swimming to find the platform, partially rectified in the APP_SWE_/PS1_ΔE9_ IFNAR1^−/−^ counterparts. Wildtype and IFNAR1^−/−^ behaved similarly (Fig. 2d, Additional file 2: Table S1 for detailed analysis).

After the 7 day acquisition period, the escape platform was removed from the maze and persistence of the mouse to escape was measured. Although not statistically significant, APP_SWE_/PS1_ΔE9_ x IFNAR1^−/−^ mice spent more time exploring the escape quadrant than APP_SWE_/PS1_ΔE9_ mice (APP_SWE_/PS1_ΔE9_: 27.3 ± 3.8 % vs. APP_SWE_/PS1_ΔE9_ x IFNAR1^−/−^: 39.0 ± 7.7 %, *n* = 9–18 per genotype, *p =* 0.5111, Fig. 2e). Interestingly, IFNAR1^−/−^ mice spent a significantly greater amount of time in the escape quadrant than APP_SWE_/PS1_ΔE9_ mice (IFNAR1^−/−^: 45.6 ± 0.5 % vs. APP_SWE_/PS1_ΔE9_: 27.3 ± 3.8 %, *n* = 9–18 per genotype, *p =* 0.0488, Fig. 2e). As swimming ability can represent a potential confounding factor in the Morris water maze, average swim speed was measured. APP_SWE_/PS1_Δ9_ mice swim at a significantly lower velocity than their wildtype counterparts (Wildtype: 18.5 ± 0.5 cm/s vs. APP_SWE_/PS1_Δ9_: 15.9 ± 0.4 cm/s, *n* = 9–18 per genotype, *p =* 0.0025, Fig. 2f); however this difference at a physiological level is minor and observed swimming technique remained consistent amongst genotypes. Collectively, this data implicates that removal of type-1 IFN signaling in APP_SWE_/PS1_ΔE9_ mice rescues spatial learning and memory deficits assessed using the Morris water maze.

### The type-1 IFN and pro-inflammatory cytokine response is attenuated in APP_SWE_/PS1_ΔE9_ x IFNAR1^−/−^ mice

**Fig. 3:**
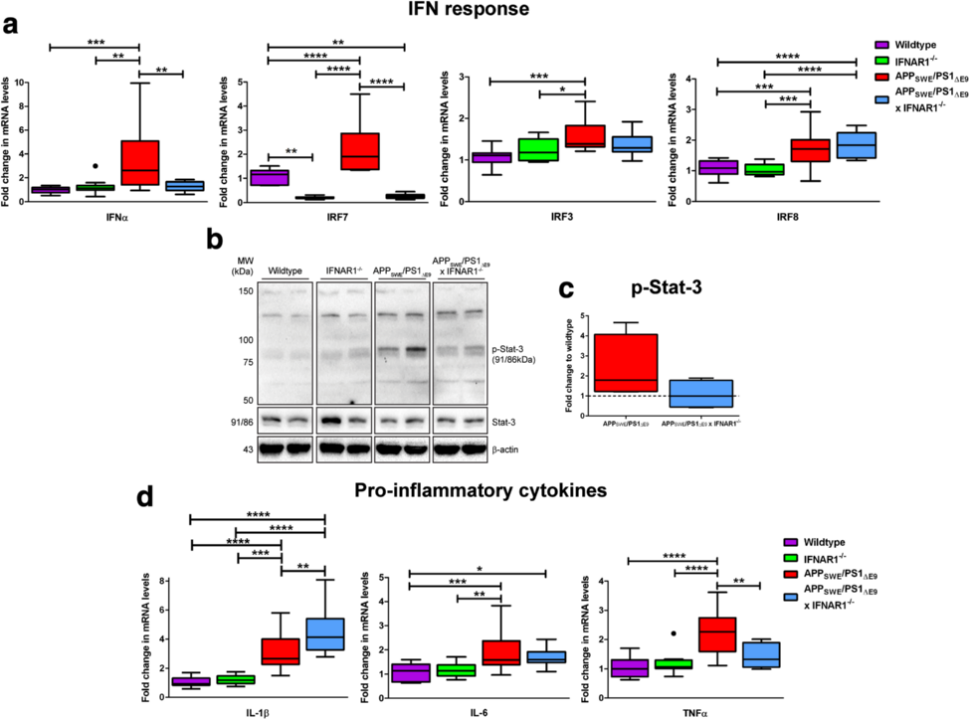
The type-1 IFN and pro-inflammatory cytokine response is attenuated upon removal of IFNAR1 in APP_SWE_/PS1_ΔE9_ mice. **a** Q-PCR of cortical tissue isolated from 9 month old wildtype, IFNAR1^−/−^, APP_SWE_/PS1_ΔE9_ and APP_SWE_/PS1_ΔE9_ x IFNAR1^−/−^ littermate controls analyzing IFNα, IRF7, IRF3 and IRF8 transcript levels. **b** Representative immunoblot of Tris–HCl soluble cortical protein lysates isolated from 9 month old wildtype, IFNAR1^−/−^, APP_SWE_/PS1_ΔE9_ and APP_SWE_/PS1_ΔE9_ x IFNAR1^−/−^ mice using anti-p-Stat-3. **c** Densitometry of cortical p-Stat-3 levels in APP_SWE_/PS1_ΔE9_ and APP_SWE_/PS1_ΔE9_ x IFNAR1^−/−^ mice is shown. **d** Q-PCR of cortical tissue isolated from 9 month old wildtype, IFNAR1^−/−^, APP_SWE_/PS1_ΔE9_ and APP_SWE_/PS1_ΔE9_ x IFNAR1^−/−^ littermate controls analyzing IL-1β, IL-6 and TNFα transcript levels. For Q-PCR, all samples were normalized back to the Ct value of the housekeeping gene GAPDH (ΔCt). The mRNA of the variant genotype groups were then expressed relative to their gene-specific wildtype littermate controls (fold change, ΔΔCt). For densitometry, total Stat-3 levels were normalized to the β-actin loading control and p-Stat-3 intensity was calculated relative to this value (p-Stat-3/(Stat-3/β-actin). Intensity values of the APP_SWE_/PS1_ΔE9_ and APP_SWE_/PS1_ΔE9_ x IFNAR1^−/−^ mouse groups are expressed as fold change relative to wildtype littermate control levels (represented by the dashed line). Immunodetection of β-actin was used to ascertain loading quantities. Data are displayed as box plots box plots described in the statistical analysis section in Materials and Methods (Q-PCR: *n* = 9 per genotype; Western blotting: *n* = 4 per genotype; •represents outlier value; *p < 0.05, **p < 0.01, ***p < 0.001, ****p < 0.0001). See Additional file 2: Table S1 for further analysis

Previously it has been demonstrated that removal of IFNAR1 attenuates the type-1 IFN response to soluble Aβ1-42 in primary cultured neurons and confers neuro-protection [64]. To investigate alterations in the type-1 IFN response in APP_SWE_/PS1_ΔE9_ x IFNAR1^−/−^ mice, Q-PCR was performed on cortical tissue. Levels of IFNα expression were significantly elevated in APP_SWE_/PS1_ΔE9_ mice compared to wildtype mice with this elevation attenuated in APP_SWE_/PS1_ΔE9_ x IFNAR1^−/−^ mice (Wildtype: 1.0 ± 0.08-fold vs. APP_SWE_/PS1_ΔE9_: 3.4 ± 0.8-fold, *p =* 0.0009; APP_SWE_/PS1_ΔE9_: 3.4 ± 0.8-fold vs. APP_SWE_/PS1_ΔE9_ x IFNAR1^−/−^: 1.3 ± 0.1-fold, *p =* 0.0063, *n* = 9 per genotype, Fig. 3a). This data confirms that aged APP_SWE_/PS1_ΔE9_ display enhanced type-1 IFNα expression that is IFNAR1-dependent. We also analyzed IFNβ transcript levels in both wildtype and APP_SWE_/PS1_ΔE9_ cortical tissue but were unable to detect a difference between genotypes (*n* = 7 per genotype, Additional file 3: Figure S2).

As IRF7 and IRF3 are critical mediators of IFNα [27] and IFNβ [56] production respectively, mRNA levels were also analyzed to assess the capacity for type-1 IFN production in these mice. Levels of IRF7 expression were significantly elevated in APP_SWE_/PS1_ΔE9_ mice compared to wildtype mice, implying elevated capacity for IFNα production in these mice (Wildtype: 1.1 ± 0.08-fold vs. APP_SWE_/PS1_ΔE9_: 2.2 ± 0.3-fold, *p <* 0.0001, *n* = 9 per genotype, Fig. 3a). This elevation in IRF7 was attenuated in APP_SWE_/PS1_ΔE9_ x IFNAR1^−/−^ mice (APP_SWE_/PS1_ΔE9_: 2.2 ± 0.3-fold vs. APP_SWE_/PS1_ΔE9_ x IFNAR1^−/−^: 0.3 ± 0.03-fold, *p <* 0.0001, *n* = 9 per genotype, Fig. 3a). Interestingly, IFNAR1^−/−^ mice exhibit basal reductions in IRF7 expression levels compared to wildtype mice (Wildtype: 1.1 ± 0.08-fold vs. IFNAR1^−/−^: 0.2 ± 0.02-fold, *p =* 0.0019, *n* = 9 per genotype, Fig. 3a). Expression levels of IRF3 were significantly elevated in APP_SWE_/PS1_ΔE9_ mice compared to wildtype mice (Wildtype: 1.1 ± 0.06-fold vs. APP_SWE_/PS1_ΔE9_: 1.5 ± 0.1-fold, *p =* 0.0004, *n* = 9 per genotype, Fig. 3a). However no alteration was detected when IRF3 levels in APP_SWE_/PS1_ΔE9_ mice were compared to APP_SWE_/PS1_ΔE9_ x IFNAR1^−/−^, implying that signaling through IFNAR1 does not regulate IRF3 expression in these mice (APP_SWE_/PS1_ΔE9_: 1.5 ± 0.1-fold vs. APP_SWE_/PS1_ΔE9_ x IFNAR1^−/−^: 1.4 ± 0.09-fold, *p =* 0.4369, *n* = 9 per genotype, Fig. 3a). We also analyzed transcript levels of IRF8, a type-1 IFN-regulated mediator important in microglial activation and phenotype [40]. Levels of IRF8 expression were significantly elevated in APP_SWE_/PS1_ΔE9_ mice compared to wildtype mice (Wildtype: 1.1 ± 0.07-fold vs. APP_SWE_/PS1_ΔE9_: 1.7 ± 0.2-fold, *p =* 0.0009, *n* = 9 per genotype, Fig. 3a) and this elevation was maintained in APP_SWE_/PS1_ΔE9_ x IFNAR1^−/−^ mice (APP_SWE_/PS1_ΔE9_: 1.7 ± 0.2-fold vs. APP_SWE_/PS1_ΔE9_ x IFNAR1^−/−^: 1.9 ± 0.1-fold, *p =* 0.3845, *n* = 9 per genotype, Fig. 3a).

Considering type-1 IFNs signal via the JAK-Stat cascade and induce pro-inflammatory cytokine transcription, phosphorylation of Stat-3 was analyzed as a reporter of net type-1 IFN signaling in the APP_SWE_/PS1_ΔE9_ x IFNAR1^−/−^ mice. Western blotting confirmed elevated phosphorylation of Stat-3 in APP_SWE_/PS1_ΔE9_ mice compared to both wildtype and IFNAR1^−/−^ mice (*n* = 4 per genotype, Fig. 3b). Densitometry of these blots identified a trend for decreased Stat-3 activation in APP_SWE_/PS1_ΔE9_ x IFNAR1^−/−^ mice compared to APP_SWE_/PS1_ΔE9_ mice (APP_SWE_/PS1_ΔE9_: 2.4 ± 0.8-fold vs. APP_SWE_/PS1_ΔE9_ x IFNAR1^−/−^: 1.1 ± 0.4-fold, *p =* 0.1955, *n* = 4 per genotype Fig. 3c). Collectively, these data highlight that removal of IFNAR1 attenuates the type-1 IFN response in aged APP_SWE_/PS1_ΔE9_ mice, correlating with cognitive benefits and modest reductions in Aβ monomer load.

Type-1 IFNs are master regulators of the innate immune response, regulating pro-inflammatory cytokine production [33]. To investigate if the removal of type-1 IFN signaling alters pro-inflammatory cytokine secretion in APP_SWE_/PS1_ΔE9_ mice, Q-PCR analyzing cortical tissue was performed. IL-1β mRNA transcript levels were upregulated in the APP_SWE_/PS1_ΔE9_ mice compared wildtype mice (Wildtype: 1.1 ± 0.09-fold vs. APP_SWE_/PS1_ΔE9_: 3.1 ± 0.4-fold, *p <* 0.0001, *n* = 9 per genotype, Fig. 3d). Interestingly, APP_SWE_/PS1_ΔE9_ x IFNAR1^−/−^ mice displayed elevated IL-1β mRNA levels compared to APP_SWE_/PS1_ΔE9_ mice alone (APP_SWE_/PS1_ΔE9_: 3.1 ± 0.4-fold vs. APP_SWE_/PS1_ΔE9_ x IFNAR1^−/−^: 4.5 ± 0.5-fold, *p =* 0.0071, *n* = 9 per genotype, Fig. 3d). Whilst IL-6 expression levels were significantly elevated in APP_SWE_/PS1_ΔE9_ mice when compared to wildtype mice, this response was not significantly altered in APP_SWE_/PS1_ΔE9_ x IFNAR1^−/−^ mice (Wildtype: 1.1 ± 0.1-fold vs. APP_SWE_/PS1_ΔE9_: 2.0 ± 0.2-fold, *p =* 0.0005, *n* = 9 per genotype, Fig. 3d). TNFα mRNA transcript levels were upregulated in the APP_SWE_/PS1_ΔE9_ mice compared wildtype mice (Wildtype: 1.0 ± 0.09-fold vs. APP_SWE_/PS1_ΔE9_: 2.2 ± 0.2-fold, *p <* 0.0001, *n* = 9 per genotype, Fig. 3d) Significantly, TNFα expression was reduced in the APP_SWE_/PS1_ΔE9_ IFNAR1^−/−^ mice compared to APP_SWE_/PS1_ΔE9_ counterparts (APP_SWE_/PS1_ΔE9_: 2.2 ± 0.2-fold vs. APP_SWE_/PS1_ΔE9_ x IFNAR1^−/−^: 1.4 ± 0.1-fold, *p =* 0.0037, *n* = 9 per genotype, Fig. 3d). These data suggest that type-1 IFN signaling through IFNAR1 is an important regulator of pro-inflammatory cytokine expression in APP_SWE_/PS1_ΔE9_ mice.

### APP_SWE_/PS1_ΔE9_ x IFNAR1^−/−^ mice exhibit enhanced astrocyte reactivity but attenuated microgliosis surrounding amyloid deposition

**Fig. 4:**
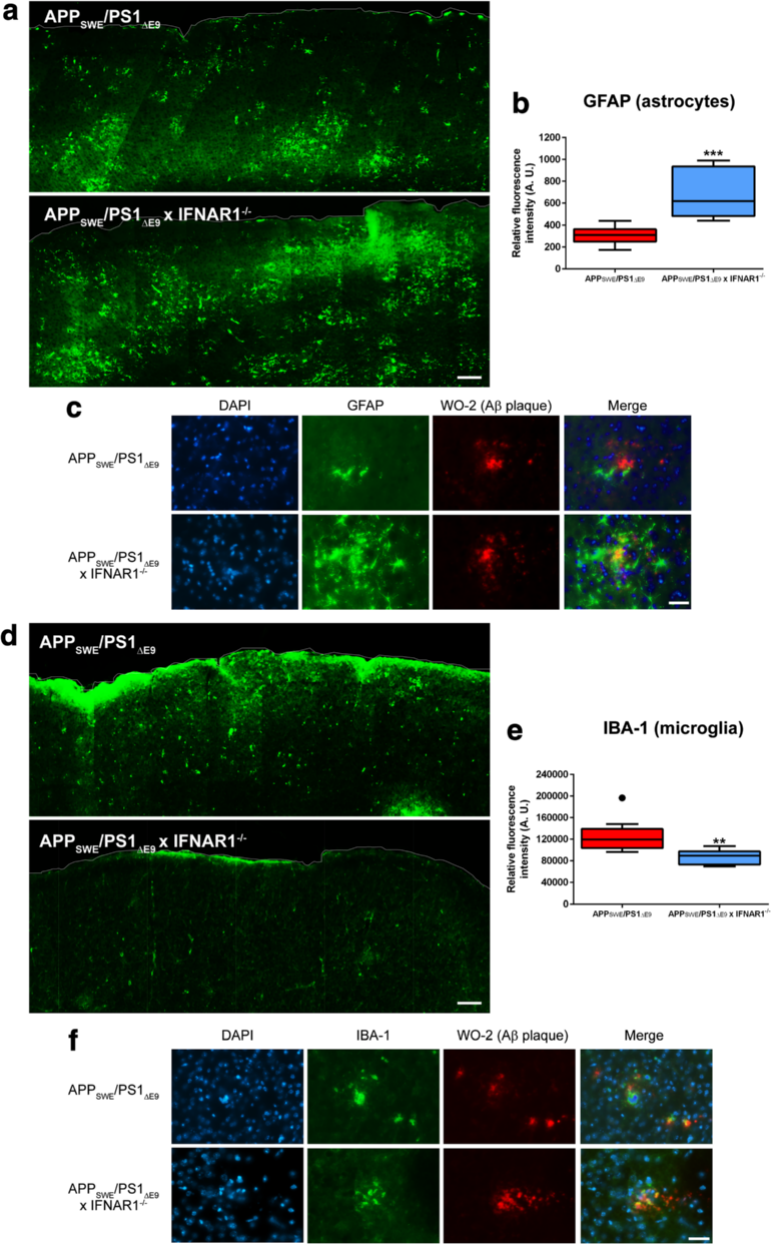
Astrocyte reactivity is elevated but microgliosis is dampened in APP_SWE_/PS1_ΔE9_ x IFNAR1^−/−^ mice. **a** Representative cortical sections from 9 month old APP_SWE_/PS1_ΔE9_ and APP_SWE_/PS1_ΔE9_ x IFNAR1^−/−^ mice stained with anti-GFAP using fluorescence immunohistochemistry. **b** Integrated density values of positive GFAP immunofluorescence were calculated from entire cortical regions of APP_SWE_/PS1_ΔE9_ and APP_SWE_/PS1_ΔE9_ x IFNAR1^−/−^ mice (3 sections per mouse). **c** High power magnification images of APP_SWE_/PS1_ΔE9_ and APP_SWE_/PS1_ΔE9_ x IFNAR1^−/−^ mouse cortical sections triple-labelled with DAPI, anti-GFAP and anti-WO-2. **d** Representative cortical sections from 9 month old APP_SWE_/PS1_ΔE9_ and APP_SWE_/PS1_ΔE9_ x IFNAR1^−/−^ mice stained with anti-IBA-1 using fluorescence immunohistochemistry. **e** Integrated density values of positive IBA-1 immunofluorescence were calculated from entire cortical regions of APP_SWE_/PS1_ΔE9_ and APP_SWE_/PS1_ΔE9_ x IFNAR1^−/−^ mice (3 sections per mouse). **f** High power magnification images of APP_SWE_/PS1_ΔE9_ and APP_SWE_/PS1_ΔE9_ x IFNAR1^−/−^ mouse cortical sections triple-labelled with DAPI, anti-IBA-1 and anti-WO-2. **b** Scale bars: low power = 200 μm; high power = 30 μm. All data is displayed as box plots described in the statistical analysis section in Materials and Methods (*n* = 9 per genotype; •represents outlier value; **p < 0.01, ***p < 0.001). See Additional file 2: Table S1 for further analysis

Both microgliosis and astrocyte reactivity are important hallmarks of the neuro-inflammation evident in AD and are primary sources of pro-inflammatory cytokine production [25]. To establish if removal of type-1 IFN signaling alters astrocyte reactivity in APP_SWE_/PS1_ΔE9_ mice, immunohistochemistry was performed. Representative images and fluorescence quantification of sagittaly sectioned cortex revealed a significant 2.2 ± 0.3-fold increase in GFAP reactivity in APP_SWE_/PS1_ΔE9_ x IFNAR1^−/−^ mice compared to APP_SWE_/PS1_ΔE9_ counterparts (*p =* 0.0006, *n* = 9 per genotype, Fig. 4a, b). High power magnification images demonstrate this elevated astrocyte reactivity surrounds Aβ plaques, generating a localized inflammatory environment (Fig. 4c). Collectively, this data highlights that removal of IFNAR1 triggers increased astrocyte reactivity in cortical areas of Aβ accumulation in APP_SWE_/PS1_ΔE9_ mice. However, further investigation is required to conclude if this is a compensatory or direct effect of removing type-1 IFN signaling in APP_SWE_/PS1_ΔE9_ mice.

To assess if ablation of type-1 IFN signaling affects microgliosis in APP_SWE_/PS1_ΔE9_ mice further immunohistochemistry was performed. Representative images and fluorescence quantification of sagittaly sectioned cortex revealed a significant 1.5 ± 0.09-fold decrease in IBA-1 reactivity in APP_SWE_/PS1_ΔE9_ x IFNAR1^−/−^ mice compared to APP_SWE_/PS1_ΔE9_ counterparts (*p =* 0.0032, *n* = 9 per genotype, Fig. 4d, e). High power magnification images demonstrate a hypertrophic and reactive microglial phenotype surrounding Aβ plaques in the APP_SWE_/PS1_ΔE9_ mice. IBA-1 positive cells detected in APP_SWE_/PS1_ΔE9_ x IFNAR1^−/−^ display decreased staining intensity and remain embedded within plaque deposition, adopting a different morphology than cells in APP_SWE_/PS1_ΔE9_ mice (Fig. 4f). These findings suggest that ablation of type-1 IFN signaling in APP_SWE_/PS1_ΔE9_ mice attenuates cortical microgliosis and alters cellular morphology within the amyloid plaque microenvironment.

### Removal of IFNAR1 shifts elevates expression of anti-inflammatory glial phenotypic markers in APP_SWE_/PS1_ΔE9_ mice

It has been suggested that pro-inflammatory microglial phenotypes enhance are largely deleterious in AD whereas anti-inflammatory microglial activity can promote beneficial inflammatory resolution [53]. We have shown that altered gliosis, decreased type-1 IFN responses and altered pro-inflammatory cytokine secretion is evident in APP_SWE_/PS1_ΔE9_ x IFNAR1^−/−^ mice, thus we were interested in assessing expression of glial inflammatory phenotypic markers. Cortical tissue from wildtype, IFNAR1^−/−^, APP_SWE_/PS1_ΔE9_ and APP_SWE_/PS1_ΔE9_ x IFNAR1^−/−^ mice were analyzed by Q-PCR for pro- and anti-inflammatory glial phenotype markers.

**Fig. 5:**
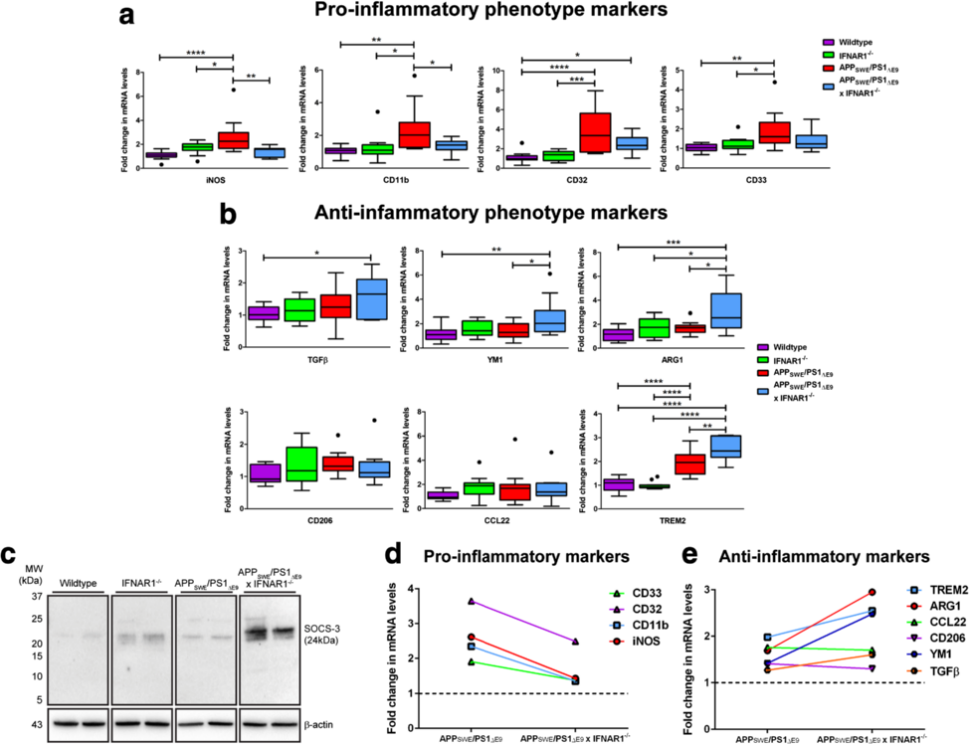
Removal of IFNAR1 in APP_SWE_/PS1_ΔE9_ mice shifts the microglial phenotype to an anti-inflammatory state. **a** Q-PCR of cortical tissue isolated from 9 month old wildtype, IFNAR1^−/−^, APP_SWE_/PS1_ΔE9_ and APP_SWE_/PS1_ΔE9_ x IFNAR1^−/−^ littermate controls analyzing iNOS, CD11b, CD32 and CD33 pro-inflammatory glial marker expression levels. **b** Q-PCR of cortical tissue isolated from 9 month old wildtype, IFNAR1^−/−^, APP_SWE_/PS1_ΔE9_ and APP_SWE_/PS1_ΔE9_ x IFNAR1^−/−^ littermate controls analyzing TGFβ, YM1, ARG1, CD206, CCL22 and TREM2 anti-inflammatory glial marker expression levels. **c** Immunoblot of Tris–HCl soluble cortical protein lysates isolated from 9 month old wildtype, IFNAR1^−/−^, APP_SWE_/PS1_ΔE9_ and APP_SWE_/PS1_ΔE9_ x IFNAR1^−/−^ mice using anti-SOCS-3. Comparative summaries of **d** Pro-inflammatory and **e** anti-inflammatory glial marker expression in APP_SWE_/PS1_ΔE9_ and APP_SWE_/PS1_ΔE9_ x IFNAR1^−/−^ mice are displayed. For Q-PCR, all samples were normalized back to the Ct value of the housekeeping gene GAPDH (ΔCt). The mRNA of the variant genotype groups were then expressed relative to their gene-specific wildtype littermate controls. For western blotting, immunodetection of β-actin was used to ascertain loading quantities. Data are displayed as mean alone or as box plots described in the statistical analysis section in Materials and Methods (*n* = 9 per genotype; •represents outlier value; *p < 0.05, **p < 0.01, ***p < 0.001, ****p < 0.0001). See Additional file 2: Table S1 for further analysis

Elevation of iNOS pro-inflammatory marker expression was confirmed in APP_SWE_/PS1_ΔE9_ mice compared to wildtype mice (Wildtype: 1.1 ± 0.1-fold vs. APP_SWE_/PS1_ΔE9_: 2.6 ± 0.4-fold, *p <* 0.0001, *n* = 9 per genotype, Fig. 5a). Significantly, iNOS expression was decreased in APP_SWE_/PS1_ΔE9_ x IFNAR1^−/−^ mice compared to APP_SWE_/PS1_ΔE9_ mice alone (APP_SWE_/PS1_ΔE9_: 2.6 ± 0.4-fold vs. APP_SWE_/PS1_ΔE9_ x IFNAR1^−/−^: 1.4 ± 0.1-fold, *p =* 0.0053, *n* = 9 per genotype, Fig. 5a). Transcript levels of the pro-inflammatory marker CD11b were elevated in APP_SWE_/PS1_ΔE9_ mice compared to wildtype mice (Wildtype: 1.0 ± 0.07-fold vs. APP_SWE_/PS1_ΔE9_: 2.4 ± 0.4-fold, *p =* 0.0014, *n* = 9 per genotype, Fig. 5a). Similar to the iNOS expression, CD11b transcript levels were decreased in APP_SWE_/PS1_ΔE9_ x IFNAR1^−/−^ mice compared to APP_SWE_/PS1_ΔE9_ mice alone (APP_SWE_/PS1_ΔE9_: 2.4 ± 0.4-fold vs. APP_SWE_/PS1_ΔE9_ x IFNAR1^−/−^: 1.4 ± 0.1-fold, *p =* 0.0371, *n* = 9 per genotype, Fig. 5a). Expression of the CD32 pro-inflammatory marker was elevated in APP_SWE_/PS1_ΔE9_ mice compared to wildtype, however this elevation was not suppressed in APP_SWE_/PS1_ΔE9_ x IFNAR1^−/−^ mice (Wildtype: 1.1 ± 0.1-fold vs. APP_SWE_/PS1_ΔE9_: 3.7 ± 0.6-fold, *p <* 0.0001, *n* = 9 per genotype, Fig. 5a). Indeed, CD32 levels in APP_SWE_/PS1_ΔE9_ x IFNAR1^−/−^ mice were elevated compared to wildtype mice albeit not to the same levels as APP_SWE_/PS1_ΔE9_ mice (Wildtype: 1.1 ± 0.1-fold vs. APP_SWE_/PS1_ΔE9_ x IFNAR1^−/−^: 2.5 ± 0.3-fold, *p =* 0.0366, *n* = 9 per genotype, Fig. 5a). Expression of the CD33 pro-inflammatory marker was elevated in APP_SWE_/PS1_ΔE9_ mice compared to wildtype, but this elevation was not suppressed in APP_SWE_/PS1_ΔE9_ x IFNAR1^−/−^ mice (Wildtype: 1.0 ± 0.05-fold vs. APP_SWE_/PS1_ΔE9_: 1.9 ± 0.3-fold, *p =* 0.0015, *n* = 9 per genotype, Fig. 5a).

Analysis of the anti-inflammatory marker TGFβ revealed elevated expression levels in APP_SWE_/PS1_ΔE9_ x IFNAR1^−/−^ mice compared to wildtype mice (Wildtype: 1.0 ± 0.06-fold vs. APP_SWE_/PS1_ΔE9_ x IFNAR1^−/−^: 1.6 ± 0.2-fold, *p =* 0.0189, *n* = 9 per genotype, Fig. 5b). This elevation was not present in the APP_SWE_/PS1_ΔE9_ cohort. Transcript levels of the YM1 anti-inflammatory marker were also elevated in APP_SWE_/PS1_ΔE9_ x IFNAR1^−/−^ mice when compared to both wildtype and APP_SWE_/PS1_ΔE9_ mice (Wildtype: 1.2 ± 0.2-fold vs. APP_SWE_/PS1_ΔE9_ x IFNAR1^−/−^: 2.5 ± 0.5-fold, *p =* 0.0061; APP_SWE_/PS1_ΔE9_: 1.4 ± 0.2-fold vs. APP_SWE_/PS1_ΔE9_ x IFNAR1^−/−^: 2.5 ± 0.5-fold, *p =* 0.0490, *n* = 9 per genotype, Fig. 5b). ARG1 anti-inflammatory marker expression levels were elevated in APP_SWE_/PS1_ΔE9_ x IFNAR1^−/−^ mice when compared to both wildtype and APP_SWE_/PS1_ΔE9_ cohorts (Wildtype: 1.2 ± 0.1-fold vs. APP_SWE_/PS1_ΔE9_ x IFNAR1^−/−^: 3.0 ± 0.5-fold, *p =* 0.0002; APP_SWE_/PS1_ΔE9_: 1.7 ± 0.2-fold vs. APP_SWE_/PS1_ΔE9_ x IFNAR1^−/−^: 3.0 ± 0.5-fold, *p =* 0.0141, *n* = 9 per genotype, Fig. 5b). Expression levels of both CD206 and CCL22 M2 markers remained constant amongst all genotypes (*n* = 9 per genotype, Fig. 5b). Transcript levels of the TREM2 anti-inflammatory marker were elevated in APP_SWE_/PS1_ΔE9_ x IFNAR1^−/−^ mice when compared to both wildtype and APP_SWE_/PS1_ΔE9_ mice (Wildtype: 1.0 ± 0.07-fold vs. APP_SWE_/PS1_ΔE9_ x IFNAR1^−/−^: 2.5 ± 0.2-fold, *p <* 0.0001; APP_SWE_/PS1_ΔE9_: 2.0 ± 0.1-fold vs. APP_SWE_/PS1_ΔE9_ x IFNAR1^−/−^: 2.5 ± 0.2-fold, *p =* 0.0056, *n* = 9 per genotype, Fig. 5b). Of interest is the finding that elevations in TREM2 expression were not unique to the APP_SWE_/PS1_ΔE9_ x IFNAR1^−/−^ genotype but also observed in APP_SWE_/PS1_ΔE9_ mice when compared to wildtype counterparts (Wildtype: 1.0 ± 0.07-fold vs. APP_SWE_/PS1_ΔE9_: 2.0 ± 0.1-fold, *p <* 0.0001, *n* = 9 per genotype, Fig. 5b). Western blot analysis of the anti-inflammatory marker SOCS-3, a negative regulator of type-1 IFN signaling and cytokine production [61, 69], confirmed an up-regulation in APP_SWE_/PS1_ΔE9_ x IFNAR1^−/−^ mice compared to APP_SWE_/PS1_ΔE9_ counterparts. This up-regulation was also confirmed in IFNAR1^−/−^ mice alone when compared to wildtype mice (Fig. 5c).

From the summarized data depicting pro-inflammatory (Fig. 5d) and anti-inflammatory glial phenotypic marker expression (Fig. 5e), these findings implicate that removal of type-1 IFN signaling shifts the glial phenotype from a pro-inflammatory phenotype towards an anti-inflammatory and presumably neuro-protective phenotype in APP_SWE_/PS1_ΔE9_ mice.

### Removal of IFNAR1 attenuates the type-1 IFN and pro-inflammatory cytokine response in response to Aβ1-42 in primary glial cultures

**Fig. 6:**
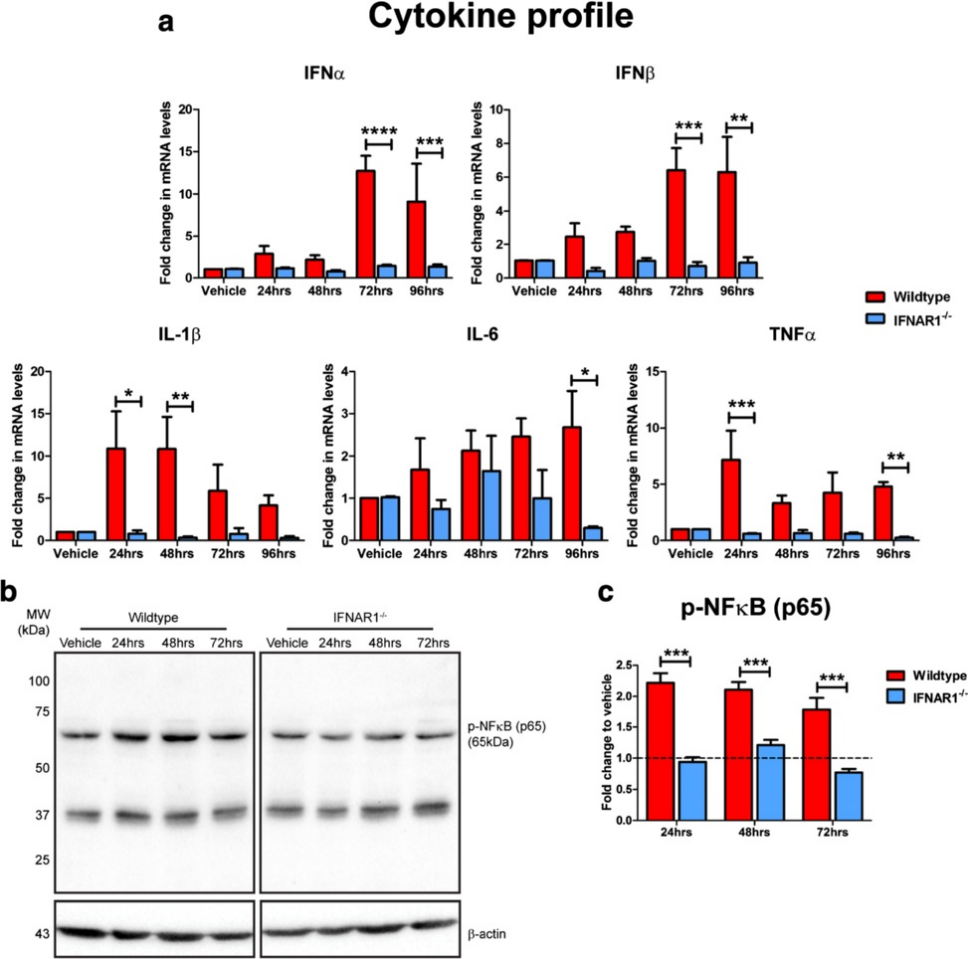
The type-1 IFN and pro-inflammatory cytokine response to Aβ1-42 is attenuated upon removal of IFNAR1 in primary cultured glia. **a** Q-PCR of primary wildtype and IFNAR1^−/−^ glial cultures treated with 10 μM Aβ1-42 for 24–96 h analyzing IFNα, IFNβ, IL-1β, IL-6 and TNFα transcript levels. **b** Representative immunoblot of primary wildtype and IFNAR1^−/−^ glial cultures treated with 10 μM Aβ1-42 for 24–72 h using anti-p-NFkB (p65). **c** Densitometry of p-NFkB (p65) levels in primary wildtype and IFNAR1^−/−^ glial cultures treated with 10 μM Aβ1-42 for 24–72 h. For Q-PCR all samples were normalized back to the Ct value of the housekeeping gene GAPDH (ΔCt). The mRNA of the Aβ1-42 treatment groups was then expressed relative to their gene-specific vehicle controls (fold change, ∆∆Ct). For densitometry raw intensities of p-NFkB (p65) bands were normalized to β-actin levels. All intensity values of Aβ1-42 treated groups are expressed as fold change relative to the genotype-specific vehicle control (average of which is represented by the dashed line). Immunodetection of β-actin was used to ascertain loading quantities. Data are displayed as mean ± SEM (Q-PCR: *n* = 4 (wildtype), *n* = 5 (IFNAR1^−/−^); Western blotting: *n* = 3 per genotype; *p < 0.05, **p < 0.01, ***p < 0.001, ****p < 0.0001). See Additional file 2: Table S1 for further analysis

Astrocytes and microglia are key contributors to the inflammatory phenotype in AD and are also sources of type-1 IFN production within the CNS [51]. To investigate the role of astroglial and microglial type-1 IFN production in response to Aβ1-42, the predominant Aβ species over-produced in APP_SWE_/PS1_ΔE9_ mice [30, 31], we adopted an in vitro approach using primary cultured mixed glial cultures. Wildtype and IFNAR1^−/−^ glia were treated with 10 μM Aβ1-42 for 24–96 h and Q-PCR was used to assess IFNα and IFNβ expression. At 72 and 96 h post-treatment IFNAR1^−/−^ glia displayed reduced IFNα (72 h: Wildtype: 12.7 ± 1.8-fold vs. IFNAR1^−/−^: 1.4 ± 0.1-fold, *p <* 0.0001; 96 h: Wildtype: 9.1 ± 4.4-fold vs. IFNAR1^−/−^: 1.3 ± 0.2-fold, *p =* 0.0007, *n* = 4–5 per genotype, Fig. 6a) and IFNβ expression (72 h: Wildtype: 6.4 ± 1.3-fold vs. IFNAR1^−/−^: 0.7 ± 0.2-fold, *p =* 0.0006; 96 h: Wildtype: 6.3 ± 2.1-fold vs. IFNAR1^−/−^: 0.9 ± 0.3-fold, *p =* 0.0012, *n* = 4–5 per genotype, Fig. 6a) compared to wildtype cultures. In contrast to our in vivo data, Western blotting and subsequent densitometry revealed that Aβ1-42 treatment did not induce a p-Stat-3 response in either wildtype or IFNAR1^−/−^ glial cultures, displaying a comparable expression level (Additional file 4: Figure S3). Overall these findings identify a glial-derived type-1 IFN response to Aβ1-42. Furthermore this type-1 IFN response is attenuated upon removal of IFNAR1, in line with the notion that IFNAR1 is critical in autocrine up-regulation of type-1 IFNs in response to inflammatory stimuli [13, 29].

To investigate if ablation of type-1 IFN signaling decreases the pro-inflammatory cytokine burden in Aβ1-42-treated glial cultures, further Q-PCR analysis was conducted. At 24 and 48 h post-treatment, the IL-1β response to Aβ1-42 was decreased in IFNAR1^−/−^ cultures compared to wildtype glia (24 h: Wildtype: 10.9 ± 4.4-fold vs. IFNAR1^−/−^: 0.8 ± 0.4-fold, *p =* 0.0105; 48 h: Wildtype: 10.8 ± 3.8-fold vs. IFNAR1^−/−^: 0.4 ± 0.1-fold, *p =* 0.0074, *n* = 4–5 per genotype, Fig. 6a). Wildtype glia generated an elevated IL-6 response upon Aβ1-42 insult that was attenuated in IFNAR1^−/−^ cultures at 96 h (Wildtype: 2.7 ± 0.9-fold vs. IFNAR1^−/−^: 0.3 ± 0.04-fold, *p =* 0.0259, *n* = 4–5 per genotype, Fig. 6a). Expression of TNFα after 24 and 96 h of Aβ1-42 treatment was also reduced in IFNAR1^−/−^ glia compared to wildtype counterparts (24 h: Wildtype: 7.2 ± 2.6-fold vs. IFNAR1^−/−^: 0.6 ± 0.05-fold, *p =* 0.0003; 96 h: Wildtype: 4.8 ± 0.4-fold vs. IFNAR1^−/−^: 0.2 ± 0.09-fold, *p =* 0.0034, *n* = 4–5 per genotype, Fig. 6a). These data suggest that type-1 IFN signaling regulates further pro-inflammatory cytokine production in glial cells exposed to Aβ1-42.

Type-1 IFNs can regulate the activity of NFkB, which is required for robust immune responses [50, 65]. To ascertain if attenuation of the type-1 IFN and pro-inflammatory cytokine response to Aβ1-42 observed in IFNAR1^−/−^ glial cultures resulted in reduced NFkB (p65) activation, further western blotting was performed (*n* = 3 per genotype, Fig. 6b). Densitometry quantification identified phosphorylation of NFkB (p65) was decreased in Aβ1-42-treated IFNAR1^−/−^ glia across the entire treatment course when compared to wildtype cultures (24 h: Wildtype: 2.2 ± 0.2-fold vs. IFNAR1^−/−^: 0.9 ± 0.08-fold, *p =* 0.0001; 48 h: Wildtype: 2.1 ± 0.1-fold vs. IFNAR1^−/−^: 1.2 ± 0.08-fold, *p =* 0.0007; 72 h: Wildtype: 1.8 ± 0.2-fold vs. IFNAR1^−/−^: 0.8 ± 0.06-fold, *p =* 0.0002, *n* = 3 per genotype, Fig. 6c). Collectively this data suggests that type-1 IFN signaling regulates the pro-inflammatory glial response to Aβ1-42.

### Wildtype glia adopt a pro-inflammatory phenotype in response to Aβ1-42, whereas IFNAR1^−/−^ cultures display enhanced expression of anti-inflammatory phenotypic markers

Within the current study we have demonstrated that removal of IFNAR1 confers an anti-inflammatory glial response in APP_SWE_/PS1_ΔE9_ mice. Thus we were interested in confirming this phenotype in Aβ1-42-treated IFNAR1^−/−^ glial cultures that display attenuated pro-inflammatory responses. To analyze the polarization phenotype in response to Aβ1-42, wildtype and IFNAR1^−/−^ glial cultures were treated with 10 μM Aβ1-42 for 24–96 h and analyzed by Q-PCR.

**Fig. 7:**
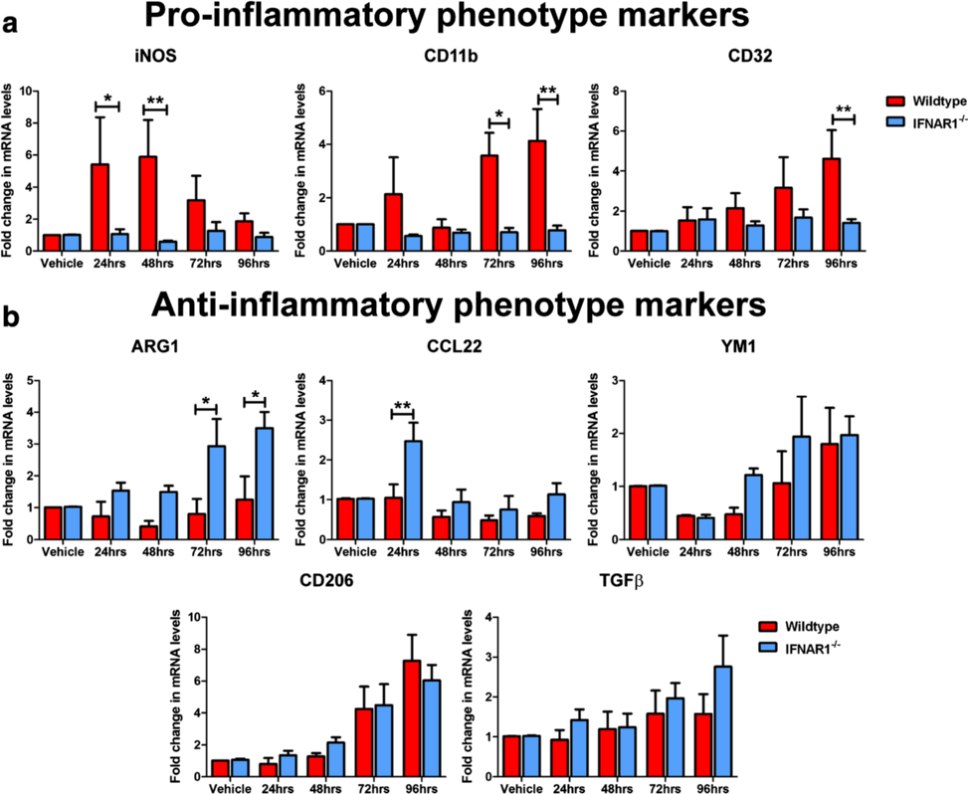
Aβ1-42 induces a pro-inflammatory phenotype in primary cultured glia with removal of IFNAR demonstrating a predmoninatly anti-inflammatory response. **a** Q-PCR of primary wildtype and IFNAR1^−/−^ glial cultures treated with 10 μM Aβ1-42 for 24–96 h analyzing iNOS, CD11b and CD32 pro-inflammatory glial marker expression levels. **b** Q-PCR of primary wildtype and IFNAR1^−/−^ glial cultures treated with 10 μM Aβ1-42 for 24–96 h analyzing ARG1, CCL22, YM1, CD206 and TGFβ anti-inflammatory glial marker expression levels. For Q-PCR all samples were normalized back to the Ct value of the housekeeping gene GAPDH (ΔCt). The mRNA of the Aβ1-42 treatment groups was then expressed relative to their gene-specific vehicle controls (fold change, ΔΔCt). Data are displayed as mean ± SEM (*n* = 4 (wildtype), *n* = 5 (IFNAR1^−/−^); *p < 0.05, **p < 0.01). See Additional file 2: Table S1 for further analysis

Significantly, expression of the iNOS pro-inflammatory marker was elevated in wildtype but not IFNAR1^−/−^ glial cultures in response to Aβ1-42 (24 h: Wildtype: 5.4 ± 2.8-fold vs. IFNAR1^−/−^: 1.1 ± 0.3-fold, *p =* 0.0195; 48 h: Wildtype: 5.9 ± 2.3-fold vs. IFNAR1^−/−^: 0.6 ± 0.06-fold, *p =* 0.0032, *n* = 4–5 per genotype, Fig. 7a). Transcript levels of the CD11b pro-inflammatory marker were also elevated in Aβ1-42-treated wildtype cultures but not when IFNAR1 was absent (72 h: Wildtype: 3.6 ± 0.9-fold vs. IFNAR1^−/−^: 0.7 ± 0.2-fold, *p =* 0.0274; 96 h: Wildtype: 4.1 ± 1.2-fold vs. IFNAR1^−/−^: 0.8 ± 0.2-fold, *p =* 0.0083, *n* = 4–5 per genotype, Fig. 7a). Expression levels of the CD32 pro-inflammatory marker were also reduced in IFNAR1^−/−^ glial cultures when compared to wildtype counterparts upon Aβ1-42 insult (96 h: Wildtype: 4.6 ± 1.4-fold vs. IFNAR1^−/−^: 1.4 ± 0.2-fold, *p =* 0.0054, *n* = 4–5 per genotype, Fig. 7a).

Analysis of the ARG1 anti-inflammatory marker revealed elevated expression levels in Aβ1-42-treated IFNAR1^−/−^ glia but not wildtype cultures (72 h: Wildtype: 0.8 ± 0.5-fold vs. IFNAR1^−/−^: 2.9 ± 0.9-fold, *p =* 0.0252; 96 h: Wildtype: 1.3 ± 0.7-fold vs. IFNAR1^−/−^: 3.5 ± 0.5-fold, *p =* 0.0163, *n* = 4–5 per genotype, Fig. 7b). CCL22 anti-inflammatory marker expression levels were also elevated in Aβ1-42-treated IFNAR1^−/−^ glia but not wildtype cultures (24 h: Wildtype: 1.0 ± 0.3-fold vs. IFNAR1^−/−^: 2.5 ± 0.5-fold, *p =* 0.0089, *n* = 4–5 per genotype, Fig. 7b). Expression levels of the anti-inflammatory markers YM1 and TGF-β remained constant across all time points and between genotypes (*n* = 4–5 per genotype, Fig. 7b). Expression levels of the CD206 anti-inflammatory marker were elevated in response to Aβ1-42 treatment but no difference between culture genotype was detected (*n* = 4–5 per genotype, Fig. 7b). Expression levels of the TGFβ anti-inflammatory marker remained constant across all time points and between genotypes (*n* = 4–5 per genotype, Fig. 7b). Collectively these data suggest that wildtype glia adopt a mixed inflammatory polarization phenotype in response to amyloid insult. Removal of IFNAR1 shifts this mixed population towards a predominantly anti-inflammatory polarization state.

### Conditioned media from Aβ1-42-treated IFNAR1^−/−^ primary glia is less toxic to primary cultured neurons than wildtype media

**Fig. 8:**
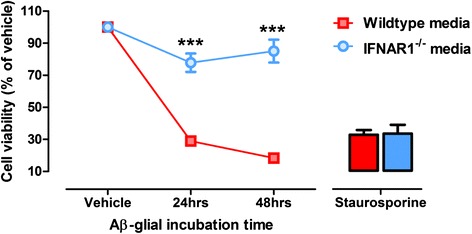
Aβ1-42-conditioned media from IFNAR1^−/−^ glia induces less neurotoxicity than wildtype counterparts. Primary wildtype and IFNAR1^−/−^ glial cultures were treated with 10 μM Aβ1-42 for 24–48 h and conditioned media was transferred to primary cultured wildtype neurons. An MTS assay was performed to assess cellular viability of neuronal cultures. Apoptosis-inducing staurosporine treatment was utilized as a cytotoxic positive control. Data are displayed as mean ± SEM (*n* = 3 per genotype (independent glial and neuronal primary cultures); ***p < 0.001). See Additional file 2: Table S1 for further analysis

To investigate the contribution of the glial polarized inflammatory response to Aβ1-42 on neuronal viability, primary wildtype and IFNAR1^−/−^ mixed glial cultures were treated with 10 μM Aβ1-42 for 24–48 h and media was collected. Primary wildtype neuronal cultures were then supplemented with this media for 48 h and an MTS assay was performed to assess cellular viability. Significantly, treatment of neurons with wildtype glial conditioned media induced severe cytotoxicity that was attenuated when the same neurons were supplemented with IFNAR1^−/−^ glial conditioned media (24 h media: Wildtype: 28.9 ± 1.2 % vs. IFNAR1^−/−^: 77.8 ± 5.7 %, *p =* 0.0003; 48 h media: Wildtype: 18.4 ± 1.9 % vs. IFNAR1^−/−^: 85.1 ± 7.1 %, *p =* 0.0001, *n* = 3 individual neuronal and glial cultures per genotype, Fig. 8). Both genotypes showed equal susceptibility to staurosporine-induced apoptosis. This data implies that the reduced Aβ1-42-induced pro-inflammatory cytokine burden and anti-inflammatory activity identified in IFNAR1^−/−^ glia is protective to neurons in vitro.

## Discussion

**Fig. 9:**
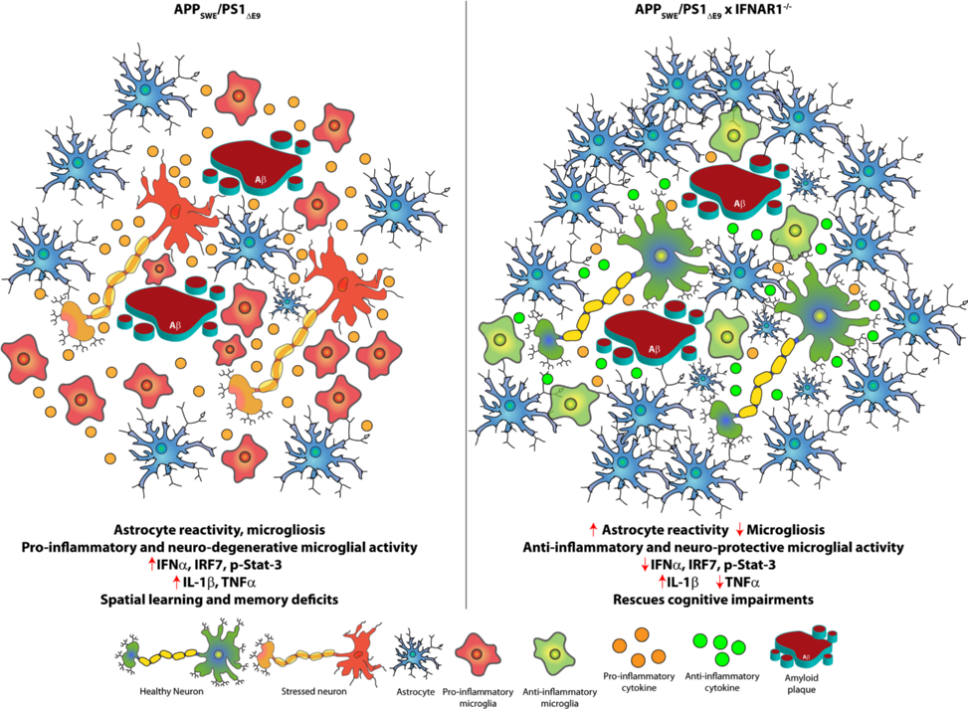
Schematic of the modulated neuro-inflammatory environment in APP_SWE_/PS1_ΔE9_ x IFNAR1^−/−^ mice. The current study provides evidence that a type-1 IFN response contributes to the neuro-inflammation observed in AD. Amyloid plaques and soluble Aβ1-42 within the plaque microenvironment triggers pro-inflammatory microglial activation and secretion of pro-inflammatory cytokines, initiating the neuro-inflammatory process. The pro-inflammatory cues within the plaque microenvironment further enhance gliosis, exacerbating inflammation. In AD, excessive Aβ production maintains the stimulus for a pro-inflammatory response, compromising resolution, and contributes to a self-perpetuating neuro-degenerative inflammatory cycle. The current study demonstrates that type-1 IFN signaling intricately controls this neuro-inflammation. Removal of IFNAR1 in APP_SWE_/PS1_ΔE9_ mice reduced type-1 IFN production, TNFα expression and conferred an anti-inflammatory and neuro-protective anti-inflammatory activation state of microglia. Enhanced astrocyte reactivity and IL-1β expression, but decreased total microgliosis, was also demonstrated in these mice which were protected from spatial learning and memory deficits at 9 months of age

The precise mechanism of how Aβ drives neurotoxicity and exacerbation of AD remains largely unknown. Neuro-inflammation has been routinely implicated in AD and is gaining credence as a major facilitator of disease progression [25, 41, 46, 47]. Type-1 IFNs are master regulators of the innate immune response [33] regulating IL-1β, IL-6 and TNFα cytokine secretion that remain up-regulated in AD [42, 43]. The present study was designed to test the hypothesis that type-1 IFN signalling influences neuro-inflammation and subsequent pathology in the APP_SWE_/PS1_ΔE9_ mouse model of AD. To address this hypothesis we generated APP_SWE_/PS1_ΔE9_ x IFNAR1^−/−^ mice lacking type-1 IFN signaling. We identified that these mice were protected from spatial learning and memory deficits demonstrated by APP_SWE_/PS1_ΔE9_ mice. Interestingly, this phenotypic rescue did not correlate with alterations in Aβ plaque burden and only modest reductions in soluble cortical Aβ monomers were detected. Additionally, removal IFNAR1 in the APP_SWE_/PS1_ΔE9_ mouse promoted cortical astrocyte reactivity, decreased total microgliosis, and conferred a largely anti-inflammatory glial phenotype. These findings were corroborated with IFNAR1^−/−^ glial cultures initiating a predominantly anti-inflammatory response to in vitro Aβ1-42 insult (Fig. 9).

Our observation that removal of IFNAR1 in APP_SWE_/PS1_ΔE9_ mice alters many aspects of the neuro-inflammatory response, improves performance in the MWM behavioural test paradigm, but does not significantly alter amyloid pathology is notable. Whilst it is clear that amelioration of amyloidosis in the majority of preclinical AD models results cognitive benefit, we speculate that alleviating the pro-inflammatory burden on the CNS alone is sufficient to rescue at least some of the cognitive impairment demonstrated in these models. We demonstrate that IFNα and IRF7 are up-regulated in APP_SWE_/PS1_ΔE9_ mice and this expression is attenuated upon removal of IFNAR1. Crucially, IRF7 is considered a central mediator of the deleterious type-1 IFN response on neurogenesis and cognition in old mice that lack amyloid deposition; a phenotype rescued upon anti-IFNAR1 monoclonal antibody treatment [3]. Thus modulation of the type-1 IFN signalling system and subsequent neuro-inflammatory responses may be sufficient in providing cognitive benefit irrespective of the clearance of Aβ. However, we cannot rule out the modest, albeit significant, reduction in Aβ monomer levels observed in the APP_SWE_/PS1_ΔE9_ x IFNAR1^−/−^ mice and its potential effect on cognition. Indeed, targeted removal of soluble Aβ production in aged tetracycline-inducible APP_SWE_ (Tg2576) mice reverts spatial learning and memory impairments in the MWM without affecting plaque burden [21]. Thus further studies involving mass spectrometry profiles and electron microscopy of Aβ species produced in APP_SWE_/PS1_ΔE9_ x IFNAR1^−/−^ may provide insight on the mechanisms by which type-1 IFN signalling potentially influences Aβ oligomerization and cognition.

The identification of attenuated plaque-localised microgliosis, a predominant anti-inflammatory glial phenotype combined with enhanced plaque-localised astrocytic reactivity in APP_SWE_/PS1_ΔE9_ x IFNAR1^−/−^ mice is also of interest. We confirm downregulated expression of pro-inflammatory (iNOS, CD11b and CD33) and up-regulations of anti-inflammatory glial phenotypic markers (TGFβ, YM1, ARG1 and TREM2) upon removal of IFNAR1 in APP_SWE_/PS1_ΔE9_ mice. Many studies suggest that this glial phenotypic shift results in enhanced Aβ phagocytosis and clearance, yet we do not observe this. One possible explanation is that the anti-inflammatory microglial activity may be counteracted by enhanced astrocyte reactivity and elevated IL-1β secretion, known to promote amyloidosis in APP_SWE_/PS1_ΔE9_ mice [26]. Indeed type-1 IFNs are pleiotropic in nature and induce cell-type specific functions [49]. Whilst this study focuses on the pro-inflammatory role of these cytokines it is equally feasible that type-1 IFNs are also exhibiting beneficial anti-inflammatory activity in specific cell types. There are currently 14 known IFNα subtypes produced in mice, 13 in humans and a singular IFNβ isoform, that in the majority of cases require IFNAR1 for signalling. Thus the mixed inflammatory phenotype we observe in our global IFNAR1 knockout approach in APP_SWE_/PS1_ΔE9_ mice is likely due to signalling elimination of these type-1 IFN subtypes and their pleiotropic effects in multiple cell types. Further studies identifying specific type-1 IFN subtypes and their contribution to neuro-inflammatory cascades and potential impact on amyloidosis will be beneficial in understanding the progression of AD.

Stimulation of primary mixed glial cultures lacking IFNAR1 with Aβ1-42 results in a predominantly anti-inflammatory response as observed in vivo. We demonstrate that the IFNAR1^−/−^ glial response to Aβ1-42 challenge is neuro-protective compared to the wildtype pro-inflammatory response in our conditioned media paradigm. We propose two alternative explanations for the protection observed in this assay: 1) IFNAR1^−/−^ glia are more effective at removing Aβ1-42 from the media than wildtype cultures, resulting in less Aβ1-42 transfer to neuronal cultures and subsequent reduction in neuro-toxicity. 2) Aβ1-42 insult triggers a reduced pro-inflammatory response from IFNAR1^−/−^ glia compared to wildtype cultures, meaning that cytokine concentrations are reduced when transferred onto neurons resulting in neuroprotection. Indeed, the phenotype observed may be resultant from a combination of these two explanations and warrants further experimentation to explore this neuro-protective mechanism.

Whilst the use of a global IFNAR1^−/−^ mouse remains a strength of the current study, enabling conclusions based on the effect of complete removal of type-1 IFN signaling, this also remains a limitation as the central and peripheral cellular contribution to neuro-inflammation in APP_SWE_/PS1_ΔE9_ mice cannot be separated. Indeed, peripheral T-regulatory FoxP3^+ve^ cells appear to breach the CSF-blood brain barrier and their presence within the CNS impacts AD pathology [4]. Considering the important role of type-1 IFN signaling in T-cell activity [16], analyzing these cell types and other peripherally invading immune cells will be crucial in further deciphering the role of type-1 IFN signaling in progression of AD. In addition, it will be important to decipher the impact of reduced endogenous murine APP levels in APP_SWE_/PS1_ΔE9_ x IFNAR1^−/−^ mice, although endogenous Aβ production appears not to influence pathology in mice carrying both mutant *APP* and *PS1* alleles [39].

## Conclusion

The mechanisms by which neuro-neuroinflammation contributes to AD exacerbation and progression is highly complex and far from being fully understood. We provide evidence that removal of type-1 IFN signalling in the APP_SWE_/PS1_ΔE9_ mouse model of AD confers a predominantly anti-inflammatory glial response and protects from cognitive decline. However our finding that this phenotype does not correlate with alterations in amyloid deposition and only a modest reduction in Aβ monomer levels requires further investigation. Deciphering the exact contribution of type-1 IFN isoforms and cell types involved in the neuro-inflammatory response will benefit our understanding of AD pathogenesis and enhance our ability to target type-1 IFN signalling in numerous neuro-inflammatory disorders.

## Abbreviations

Aβ, amyloid-beta; AD, Alzheimer’s disease; APP, amyloid precursor protein; CTF, c-terminal fragment; ELISA, enzyme-linked immunosorbent assay; GFAP, glial fibrillary acidic protein; IBA-1, ionized calcium-binder adapter molecule-1; IFN, interferon; IFNAR1, type-1 interferon alpha receptor; IL, interleukin; IRF, interferon regulatory factor; JAK, janus-associated kinase; MTS, 3-(4,5-dimethylthiazol-2-yl)-5-(3-carboxymethoxyphenyl)-2-(4-sulfophenyl)-2H-tetrazolium; MWM, Morris water maze; NFkB, nuclear factor kappa-B; NLRP, nod-like receptor; PS1, presenilin-1; Q-PCR, quantitative-polymerase chain reaction; Stat, signal transducer and activator of transcription; TNF, tumor necrosis factor

## Additional files

Additional file 1: Figure S1. Generation and confirmation of the APP_SWE_/PS1_ΔE9_ x IFNAR1^−/−^ mouse. A) To generate a colony of C57BL/6 APP_SWE_/PS1_ΔE9_ x IFNAR1^−/−^ mice with appropriate IFNAR1^−/−^ littermate controls, heterozygous APP_SWE_/PS1_ΔE9_ mice were bred to mice homozygous for the IFNAR1 gene disruption (neomycin insert; IFNAR1^−/−^). The subsequent litters were termed F1 progeny and were expected to yield mice heterozygous for both the APP_SWE_/PS1_ΔE9_ mutation and IFNAR1-neoE5 allele (25 % male and 25 % female according to Mendelian inheritance). Upon genetic confirmation, APP_SWE_/PS1_ΔE9_ (het) x IFNAR1^+/−^ mice were then interbred to generate the first APP_SWE_/PS1_ΔE9_ x IFNAR1^−/−^ mice from F2 progeny (6.25 % male and 6.25 % female according to Mendelian inheritance). APP_SWE_/PS1_ΔE9_ (het) x IFNAR1^−/−^ mice were then interbred to generate F3 progeny consisting of APP_SWE_/PS1_ΔE9_ x IFNAR1^−/−^ (75 %) and IFNAR1^−/−^ (25 %) littermates. B) Genotyping results for the initial progeny containing APP_SWE_/PS1_ΔE9_ x IFNAR1^−/−^ and littermate IFNAR1^−/−^ mice is displayed. Genotyping PCR was performed using a combined APP_SWE_/PS1_ΔE9_ and IFNAR1^−/−^. For each mouse, genotyping for APP_SWE_, PS1_ΔE9_ and IFNAR1 expression was performed in separate reactions with internal control (IC) amplification (APP_SWE_ reactions only). The expected band sizes were as follows: APP_SWE_: 377 bp, PS1_ΔE9_: 608 bp, wildtype IFNAR1: 351 bp, IFNAR1-neomycin (IFNAR1^−/−^): 985 bp and IC: 324 bp. APP positive bands in lanes 2, 8 and 12 are comprised of both APP_SWE_ and IC bands as indicated in the fig. C) Immunoblotting of Tris–HCl soluble cortical brain extracts, using mAb WO-2, revealed effective APP overexpression in APP_SWE_/PS1_ΔE9_ x IFNAR1^−/−^ mice that was indistinguishable from levels in APP_SWE_/PS1_ΔE9_ mice. (TIF 6948 kb) Additional file 2: Table S1. Statistical table (See additional statistical table file). Statistical analysis information is displayed for all data displayed within the accompanying figures. Power values were determined post-hoc based upon population effect size, sample size and group number. An α value of 0.05 was used to set the type-1 error rate in statistical comparisons. *p* values are given as exact values or adjusted multiplicity variants where an ANOVA and multiple comparisons post-hoc test was applied to the data. *n* represents the number of individual animals or primary cell cultures derived from separate animals used in each experiment. N/A: not applicable. (XLSX 49 kb) Additional file 3: Figure S2. IFNβ mRNA transcript levels are unaltered in cortical tissue 9 month old APP_SWE_/PS1_ΔE9_ mice. Q-PCR of cortical tissue isolated from 9 month old wildtype and APP_SWE_/PS1_ΔE9_ mice analyzing IFNβ mRNA levels. For Q-PCR, all samples were normalized back to the Ct value of the housekeeping gene GAPDH (ΔCt). The mRNA of the variant genotype groups were then expressed relative to their gene-specific wildtype littermate controls (fold change, ΔΔCt). Data are displayed as box plots box plots described in the statistical analysis section in Materials and Methods (*n* = 7 per genotype) See Additional file 2: Table S1 for further analysis. (TIF 213 kb) Additional file 4: Figure S3. Aβ1-42 does not induce a p-Stat-3 mediated response in either wildtype or IFNAR1^−/−^ primary glial cultures. A) Representative immunoblot of primary wildtype and IFNAR1^−/−^ glial cultures treated with 10 μM Aβ1-42 for 24–72 h using anti-p-Stat-3. B) Densitometry of p-Stat-3 levels in primary wildtype and IFNAR1^−/−^ glial cultures treated with 10 μM Aβ1-42 for 24–72 h. For densitometry, total Stat-3 levels were normalized to the β-actin loading control and p-Stat-3 intensity was calculated relative to this value (p-Stat-3/(Stat-3/β-actin). All intensity values of Aβ1-42 treated groups are expressed as fold change relative to the genotype-specific vehicle control (average of which is represented by the dashed line). Immunodetection of β-actin was used to ascertain loading quantities. Data are displayed as mean ± SEM (*n* = 3 per genotype). See Additional file 2: Table S1 for further analysis. (TIF 4277 kb)
