# Supplementary figures and images for: Deletion of the type-1 interferon receptor in APPSWE/PS1ΔE9 mice preserves cognitive function and alters glial phenotype
Source: Acta Neuropathol Commun. 2016 Jul 11;4:72. doi: 10.1186/s40478-016-0341-4 (PMC4940712; doi:10.1186/s40478-016-0341-4)

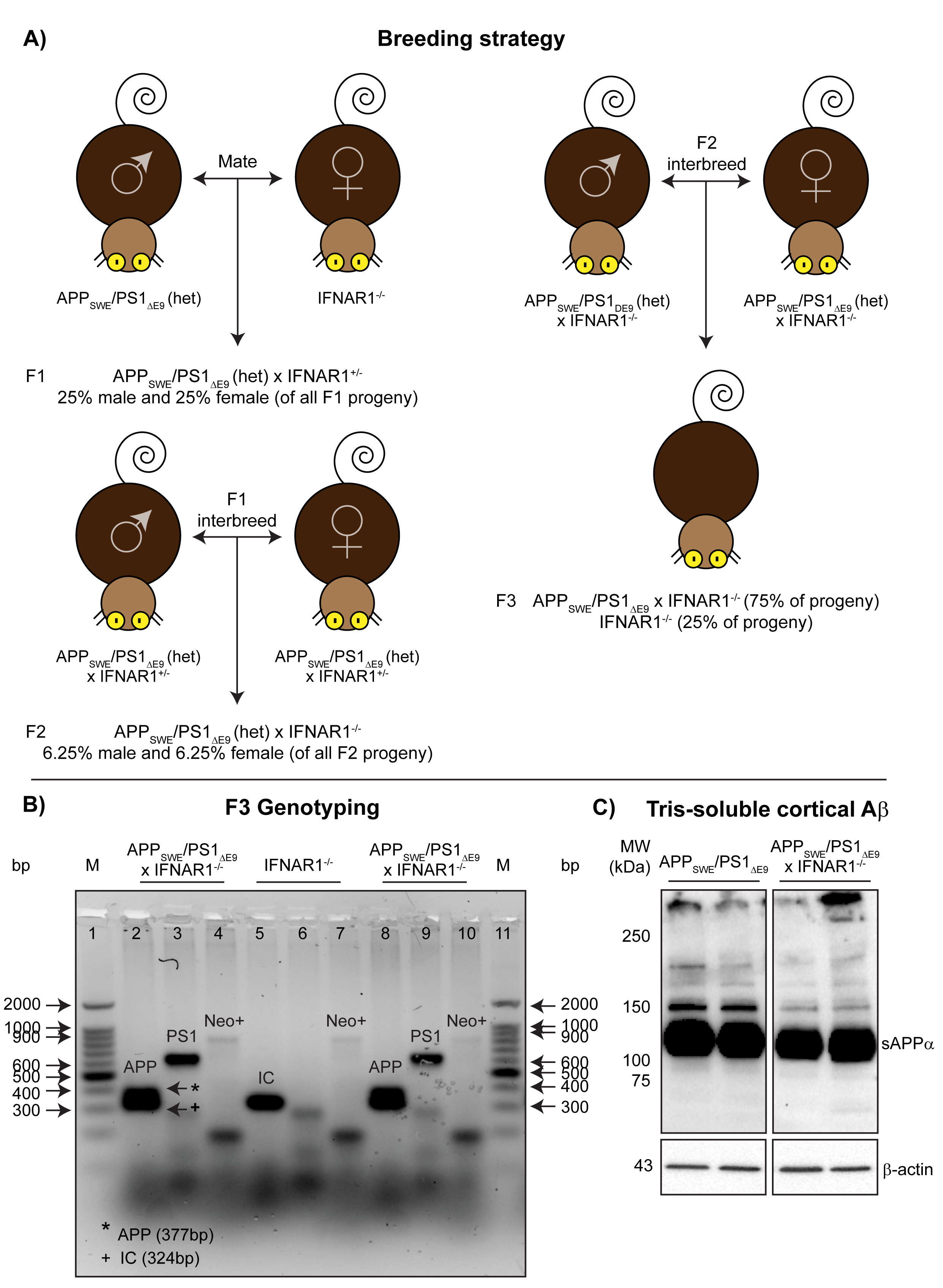

Supplement: Additional file 1: Figure S1. — Generation and confirmation of the APPSWE/PS1ΔE9 x IFNAR1−/− mouse. A) To generate a colony of C57BL/6 APPSWE/PS1ΔE9 x IFNAR1−/− mice with appropriate IFNAR1−/− littermate controls, heterozygous APPSWE/PS1ΔE9 mice were bred to mice homozygous for the IFNAR1 gene disruption (neomycin insert; IFNAR1−/−). The subsequent litters were termed F1 progeny and were expected to yield mice heterozygous for both the APPSWE/PS1ΔE9 mutation and IFNAR1-neoE5 allele (25 % male and 25 % female according to Mendelian inheritance). Upon genetic confirmation, APPSWE/PS1ΔE9 (het) x IFNAR1+/− mice were then interbred to generate the first APPSWE/PS1ΔE9 x IFNAR1−/− mice from F2 progeny (6.25 % male and 6.25 % female according to Mendelian inheritance). APPSWE/PS1ΔE9 (het) x IFNAR1−/− mice were then interbred to generate F3 progeny consisting of APPSWE/PS1ΔE9 x IFNAR1−/− (75 %) and IFNAR1−/− (25 %) littermates. B) Genotyping results for the initial progeny containing APPSWE/PS1ΔE9 x IFNAR1−/− and littermate IFNAR1−/− mice is displayed. Genotyping PCR was performed using a combined APPSWE/PS1ΔE9 and IFNAR1−/−. For each mouse, genotyping for APPSWE, PS1ΔE9 and IFNAR1 expression was performed in separate reactions with internal control (IC) amplification (APPSWE reactions only). The expected band sizes were as follows: APPSWE: 377 bp, PS1ΔE9: 608 bp, wildtype IFNAR1: 351 bp, IFNAR1-neomycin (IFNAR1−/−): 985 bp and IC: 324 bp. APP positive bands in lanes 2, 8 and 12 are comprised of both APPSWE and IC bands as indicated in the fig. C) Immunoblotting of Tris–HCl soluble cortical brain extracts, using mAb WO-2, revealed effective APP overexpression in APPSWE/PS1ΔE9 x IFNAR1−/− mice that was indistinguishable from levels in APPSWE/PS1ΔE9 mice. (TIF 6948 kb) [file 40478_2016_341_MOESM1_ESM.tif]

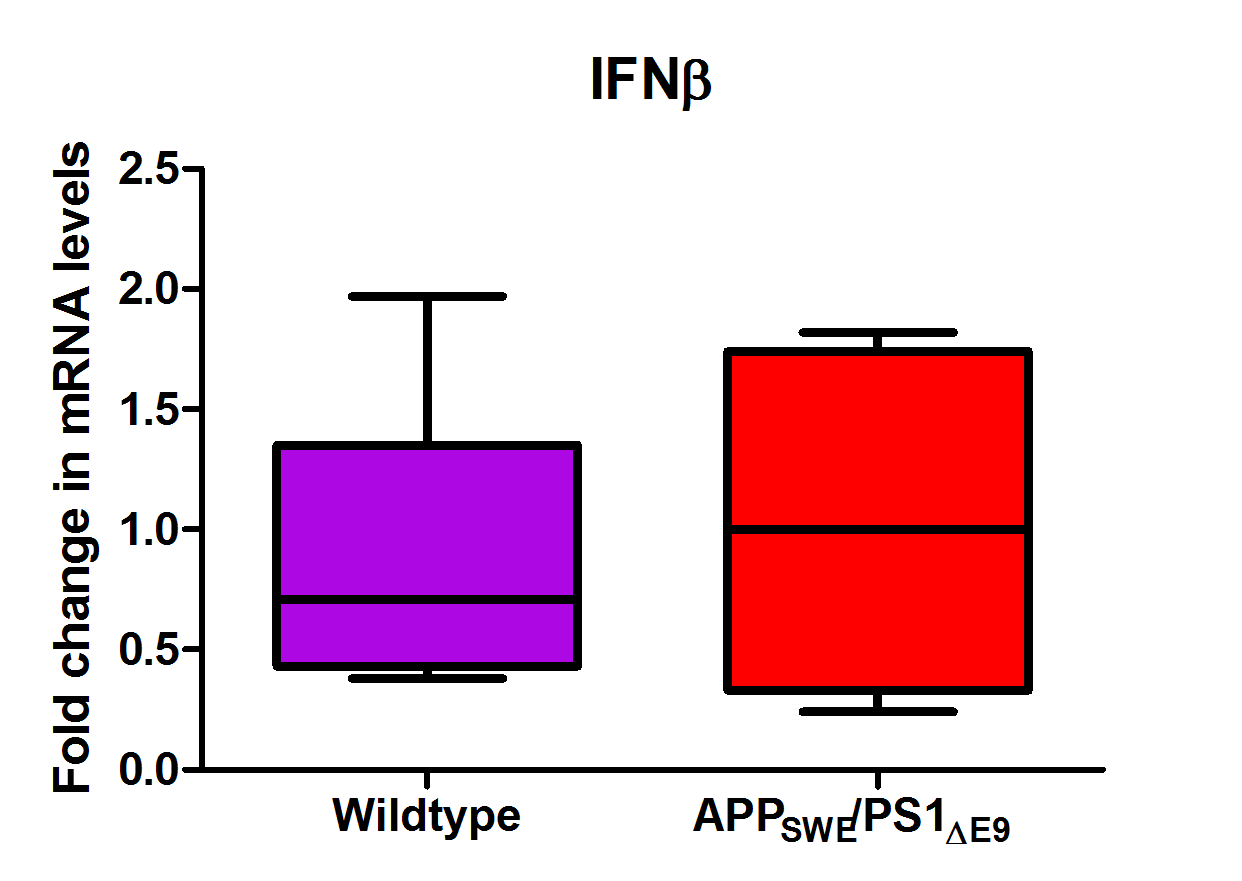

Supplement: Additional file 3: Figure S2. — IFNβ mRNA transcript levels are unaltered in cortical tissue 9 month old APPSWE/PS1ΔE9 mice. Q-PCR of cortical tissue isolated from 9 month old wildtype and APPSWE/PS1ΔE9 mice analyzing IFNβ mRNA levels. For Q-PCR, all samples were normalized back to the Ct value of the housekeeping gene GAPDH (ΔCt). The mRNA of the variant genotype groups were then expressed relative to their gene-specific wildtype littermate controls (fold change, ΔΔCt). Data are displayed as box plots box plots described in the statistical analysis section in Materials and Methods (n = 7 per genotype) See Additional file 2: Table S1 for further analysis. (TIF 213 kb) [file 40478_2016_341_MOESM3_ESM.tif]

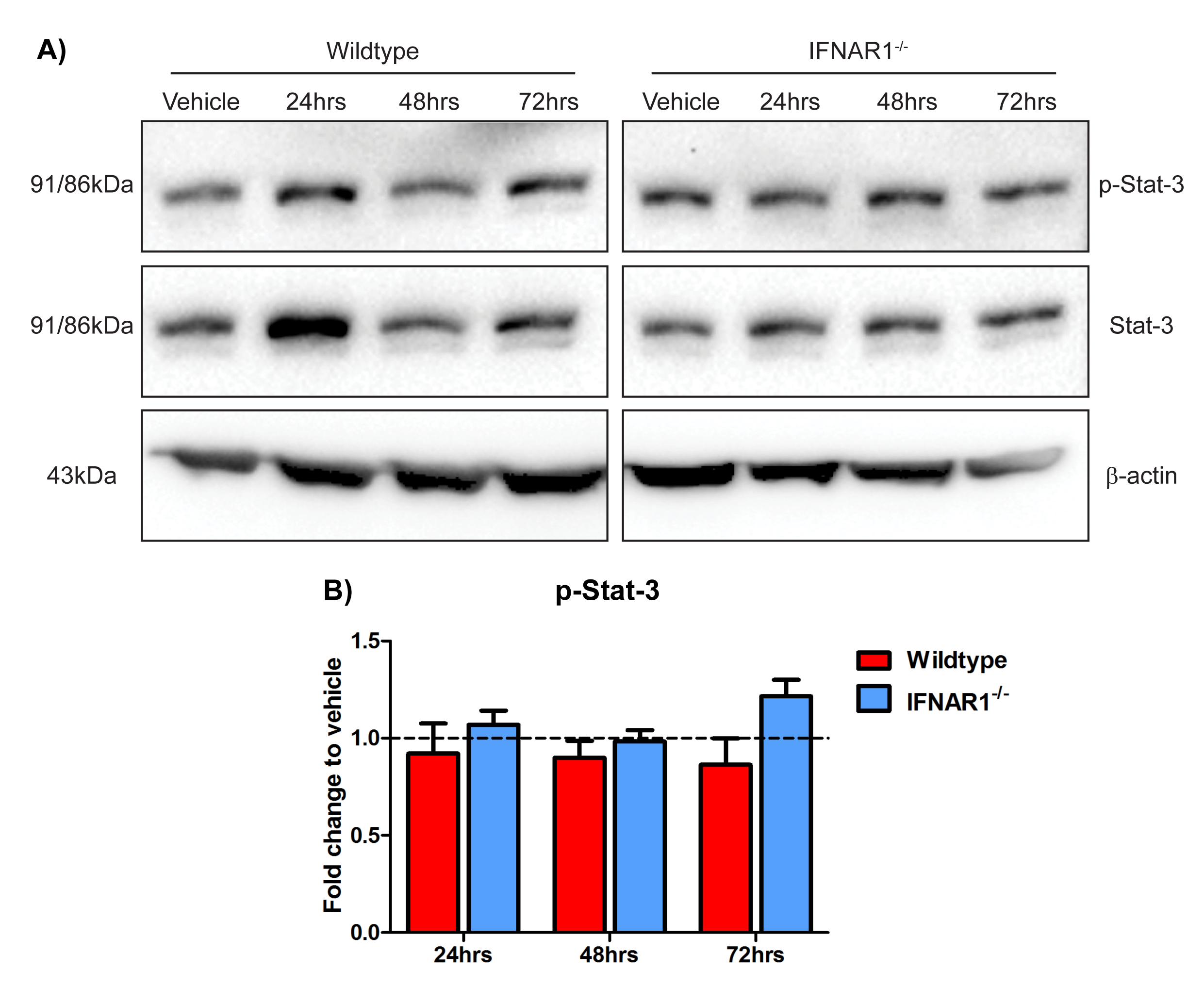

Supplement: Additional file 4: Figure S3. — Aβ1-42 does not induce a p-Stat-3 mediated response in either wildtype or IFNAR1−/− primary glial cultures. A) Representative immunoblot of primary wildtype and IFNAR1−/− glial cultures treated with 10 μM Aβ1-42 for 24–72 h using anti-p-Stat-3. B) Densitometry of p-Stat-3 levels in primary wildtype and IFNAR1−/− glial cultures treated with 10 μM Aβ1-42 for 24–72 h. For densitometry, total Stat-3 levels were normalized to the β-actin loading control and p-Stat-3 intensity was calculated relative to this value (p-Stat-3/(Stat-3/β-actin). All intensity values of Aβ1-42 treated groups are expressed as fold change relative to the genotype-specific vehicle control (average of which is represented by the dashed line). Immunodetection of β-actin was used to ascertain loading quantities. Data are displayed as mean ± SEM (n = 3 per genotype). See Additional file 2: Table S1 for further analysis. (TIF 4277 kb) [file 40478_2016_341_MOESM4_ESM.tif]
